# Does *Bidens pilosa* L. Affect Carbon and Nitrogen Contents, Enzymatic Activities, and Bacterial Communities in Soil Treated with Different Forms of Nitrogen Deposition?

**DOI:** 10.3390/microorganisms12081624

**Published:** 2024-08-09

**Authors:** Yingsheng Liu, Yizhuo Du, Yue Li, Chuang Li, Shanshan Zhong, Zhelun Xu, Congyan Wang, Daolin Du

**Affiliations:** 1 School of Environment and Safety Engineering, Jiangsu University, Zhenjiang 212013, China; 3202202017@stmail.ujs.edu.cn (Y.L.); du_yizhuo6421@163.com (Y.D.); 2222209050@stmail.ujs.edu.cn (Y.L.); 2222209081@stmail.ujs.edu.cn (C.L.); 2222109045@stmail.ujs.edu.cn (S.Z.); xzl@stu.njau.edu.cn (Z.X.); 2 Weed Research Laboratory, College of Life Sciences, Nanjing Agricultural University, Nanjing 210095, China; 3 Jiangsu Collaborative Innovation Center of Technology and Material of Water Treatment, Suzhou University of Science and Technology, Suzhou 215009, China; 4 Jingjiang College, Jiangsu University, Zhenjiang 212013, China

**Keywords:** Asteraceae, invasive plants, nitrogen deposition, soil bacterial community, soil microorganisms

## Abstract

The deposition of nitrogen in soil may be influenced by the presence of different nitrogen components, which may affect the accessibility of soil nitrogen and invasive plant–soil microbe interactions. This, in turn, may alter the success of invasive plants. This study aimed to clarify the influences of the invasive plant *Bidens pilosa* L. on the physicochemical properties, carbon and nitrogen contents, enzymatic activities, and bacterial communities in soil in comparison to the native plant *Pterocypsela laciniata* (Houtt.) Shih treated with simulated nitrogen deposition at 5 g nitrogen m^−2^ yr^−1^ in four forms (nitrate, ammonium, urea, and mixed nitrogen). Monocultural *B. pilosa* resulted in a notable increase in soil pH but a substantial decrease in the moisture, electrical conductivity, ammonium content, and the activities of polyphenol oxidase, β-xylosidase, FDA hydrolase, and sucrase in soil in comparison to the control. Co-cultivating *B. pilosa* and *P. laciniata* resulted in a notable increase in total soil organic carbon content in comparison to the control. Monocultural *B. pilosa* resulted in a notable decrease in soil bacterial alpha diversity in comparison to monocultural *P. laciniata*. Soil FDA hydrolase activity and soil bacterial alpha diversity, especially the indices of Shannon’s diversity, Simpson’s dominance, and Pielou’s evenness, exhibited a notable decline under co-cultivated *B. pilosa* and *P. laciniata* treated with nitrate in comparison to those treated with ammonium, urea, and mixed nitrogen.

## 1. Introduction

Invasive plants (IPs) have the potential to exert a considerable influence on environmental health and ecological security. In particular, IPs can affect the ecological functioning of native ecosystems, which can result in a loss of native biodiversity [1,2,3,4]. At present, there are in excess of 500 IPs distributed throughout China [5]. This is primarily attributable to the considerable diversity of habitats and climates, in addition to the recent surge in human activities, especially international trade, in China [5]. Furthermore, the Asteraceae family is the most species-rich among IPs in China, with 92 documented species to date. It constitutes ~17.86% of the total species number within IPs in China [5]. It is, therefore, crucial to elucidate the primary mechanisms underlying the successful establishment of IPS, with a particular emphasis on those observed in the Asteraceae family, as this represents a pivotal area of research within the field of invasion ecology [1,4,6,7].

One of the primary reasons for the successful colonization of IPs is their capacity to create soil microenvironments that are more conducive to further invasion through the formation of mutualistic interactions with soil microorganisms. This is accomplished through the release of a multitude of nutrients resulting from growth metabolism, litter decomposition, and root secretion, in addition to other processes. This, in turn, affects the cycling of soil nutrients, including carbon (C) and nitrogen (N), and alters the metabolic activity and community structure of soil microorganisms, thus facilitating the invasion process [8,9,10,11]. It is, therefore, of great importance to elucidate the key mechanisms by which IPS, especially the Asteraceae IPS, are successfully colonized via mutualistic interactions with soil microorganisms.

In general, N is one of the key nutritional components that restricts plant growth in several terrestrial ecosystems [12,13,14,15]. Consequently, the capacity of IPs to procure N is a pivotal determinant of their success in colonizing a diverse array of habitats. In particular, IPs are more proficient at acquiring N than native plants due to their superior levels of N availability and utilization [16,17,18,19]. Moreover, the invasion intensity and invasiveness of numerous IPs are also strongly correlated with soil N availability [20,21,22,23]. Furthermore, the invasibility of plant communities is also strictly linked to the accessibility of soil N [24,25,26]. However, the accessibility of soil N and invasive plant–soil microbe interactions may be significantly altered by atmospheric N deposition, which in turn may affect the colonization process of IPs.

The level of atmospheric N deposition has continued to increase in recent years, primarily due to the release of nitrogen-containing substances into the atmosphere from the combustion of fossil fuels in excess, the irrational or improper creation and use of nitrogenous fertilizers, the speedy progress of livestock and farming, etc. [27,28,29,30]. Presently, East Asia (principally China) has one of the three maximum levels of N deposition globally [28,31,32,33]. Nevertheless, it has been demonstrated that excessive N deposition can result in soil acidification, disturb plant growth, decrease species diversity, and significantly interrupt the ecological health and balance of ecosystems [34,35,36,37]. Moreover, N deposition can facilitate the invasion intensity and invasiveness of numerous IPs, thereby facilitating their colonization process [38,39,40,41]. It is essential to recognize that N deposition encompasses a diverse range of N components, including but not limited to NH_4_-N, NO_3_-N, and CO(NH_2_)_2_-N. The relative proportions of these components are subject to fluctuations as a consequence of alterations in energy policies and the energy mix used [42,43,44,45]. However, the deposition of N in different forms may modify the accessibility of N in soil and the interactions between IPs and soil microorganisms, which may, in turn, affect the colonization process of IPs. It is, therefore, imperative to survey the effects of N deposition with distinct N components on the available level of N in soil and the interactions between IPs and soil microorganisms. The objective of this examination is to explicate the mechanisms that facilitate the successful colonization of IPs treated with N deposition, with a particular focus on the effects of different forms of N components.

This study aimed to explicate the impacts of the Asteraceae IPs *Bidens pilosa* L. on the physicochemical properties, C and N contents, enzymatic activities, and the structure of the bacterial communities in soil (SBC) in comparison to those of the Asteraceae native plant *Pterocypsela laciniata* (Houtt.) Shih. This study was conducted with simulated N deposition at 5 g N m^−2^ yr^−1^ in four forms (nitrate (NO_3_-N), ammonium (NH_4_-N), urea (CO(NH_2_)_2_-N), and a mixed N solution comprising an equal mixture of the three individual N forms). This study was carried out over a four-month period using a pot cultivation experiment. *Bidens pilosa* has its origins in the tropical Americas and was presented to China in 1857 as part of a shipment of imported crops and vegetables. Nevertheless, *B. pilosa* has been identified as a significant threat to ecosystem structure and function, particularly in terms of biodiversity loss in China. Additionally, *B. pilosa* has been designated as a significant IP in China [4,46,47,48]. Moreover, the geographic distribution of the two Asteraceae species observed in China is among the most affected by N deposition [28,31,32,33].

The following hypotheses were proposed for this study: (1) *B. pilosa* may demonstrate a more pronounced increase in C and N contents, enzymatic activities in soil, and SBC’s alpha diversity in comparison to *P. laciniata*. (2) N form is a pivotal factor influencing C and N contents, enzymatic activities in soil, SBC’s alpha diversity, and SBC’s structure.

## 2. Materials and Methods

### 2.1. Experimental Design

The target IP was identified as *B*. *pilosa*. *Pterocypsela laciniata* was selected as the native species. Seeds of *B. pilosa* and *P. laciniata* were collected from Zhenjiang (32.15–32.16° N; 119.52–119.53° E), Jiangsu, China, in October 2022. Figure S1 provides a map indicating the geographical location of the sampled zone in this study.

A pot cultivation experiment was carried out to examine the growth of *B. pilosa* and *P. laciniata*. Pasture yellow soil (manufacturer: Shenzhibei Sci. & Technol. Co., Ltd., Baishan, China; pH value: ~6.3; organic content: ≥30%; soil electrical conductivity: ≤3 ms/cm; ~3 kg/planting basin) was selected as the culture substrate. The rationale behind the utilization of pasture yellow soil was twofold: firstly, to minimize the possibility of IPs having been introduced previously, and secondly, to mitigate the risk of contamination from N deposition in the natural soil. Seeds of *B. pilosa* and *P. laciniata* were located in garden pots (top diameter 25 cm; height 16.5 cm). A total of six seedlings of *B. pilosa* and/or *P. laciniata* with uniform size and robust growth were placed in each garden pot. The treatments were as follows: (1) six seedlings of *B. pilosa* per garden pot (monocultural *B. pilosa* (BP)); (2) three seedlings of *B. pilosa* and *P. laciniata* per garden pot (co-cultivated *B. pilosa* and *P. laciniata* (BPPL)); and (3) six seedlings of *P. laciniata* per garden pot (monocultural *P. laciniata* (PL)). All garden pots were treated with simulated N deposition comprising four forms: (1) nitrate (potassium nitrate (KNO_3_, AR, ≥99%; Aladdin^®^, Shanghai, China); inorganic nitrogen); (2) ammonium (ammonium chloride (NH_4_Cl, GR, ≥99.8%; Sinopharm Chemical Reagent Co., Ltd., Shanghai, China); inorganic nitrogen); (3) urea (CO(NH_2_)_2_, BC, ≥99.5%; Sangon Biotech Co., Ltd., Shanghai, China; organic nitrogen); (4) mixed N (nitrate–ammonium–urea = 1:1:1), at 5 g N m^−2^ yr^−1^. Sterile distilled water was employed as the control treatment, with a N concentration of 0 g N L^−1^. The content of simulated N deposition in the four aforementioned forms was set to match the real content (i.e., 5 g N m^−2^ yr^−1^) of the natural N deposition in southern Jiangsu, China, as documented in previous studies [31,32,49,50]. The proportion of three individual N in mixed N was approximated to the actual proportion of natural N deposition in southern Jiangsu, China, as documented in references [42,43,51]. All possible combinations of planted types (i.e., BP, BPPL, and PL) and N forms (i.e., nitrate, ammonium, urea, and mixed N) were investigated in this present study. Three replicates were scheduled for each treatment. The seedlings of *B. pilosa* and *P. laciniata* were cultivated in a greenhouse at Zhenjiang (32.2061° N, 119.5128° E), Jiangsu, China, under natural light for ~4 months from April to July 2023.

After a period of ~4 months, three soil cores were extracted from a depth of ~2 cm around the roots of the two plant species per garden pot via the root shaking method. Subsequently, the soil cores were homogenized to create a single soil sample, with three replicates per treatment. Soil samples were screened via a 2 mm sieve to remove any impurities, thus allowing the measurement of soil physicochemical properties, soil C and N contents, soil enzymatic activities, and SBC’s structure.

### 2.2. Determination of Soil Physicochemical Properties and Soil C and N Contents

The pH, moisture, and electrical conductivity in soil were analyzed in situ via the digital meters to ascertain soil acidity (LY-601; Jiaheng Instrument Co., Ltd., Qingdao, China) and electrical conductivity (EC-8801A; Hetai Instrument Co., Ltd., Huai’an, China).

Total soil organic C and N contents, as well as soil microbial C and N contents, were all analyzed using the chloroform fumigation method [52,53]. In particular, total soil organic C and N were extracted with 0.5 mol L^−1^ K_2_SO_4_ (1:4 soil–solution) before and after a 24 h period of chloroform fumigation. The extractable organic C and N contents in the soil extracts were subsequently analyzed by using the TOC analyzer (Analytik Jena, Jena, Germany) and flow injection analyzer (Zellwegger Analytical, Milwaukee, WI, USA), respectively. Soil microbial C and N contents were calculated as the difference between the extractable organic C and N contents of non-fumigated and fumigated soils, and an efficiency factor of 0.45 was employed to correct for incomplete extraction [52,53,54,55].

Soil ammonium and nitrate contents were analyzed by using the potassium chloride leaching method in accordance with the national standard of China (GB/T 42485-2023) [56]. In particular, soil ammonium and nitrate were extracted with 1 mol L^−1^ KCl (1:5 soil–solution) over a period of 1 h. The extractable ammonium and nitrate contents in the soil extracts were then analyzed spectrophotometrically at 543 nm and 630 nm, respectively.

### 2.3. Determination of Soil Enzymatic Activities

Several soil enzymatic activities were investigated, with a particular focus on those that affect soil C and N cycling. The following methods were employed: soil polyphenol oxidase activity was determined via the Assay Kit of Polyphenol Oxidase Activity (No.: D799508-0100; spectrophotometrically at 430 nm); soil cellulase activity was determined via the Assay Kit of Soil Cellulase Activity (No.: D799514-0100; spectrophotometrically at 540 nm); soil β-glucosidase activity was determined via the Assay Kit of Soil β-glucosidase Activity (No.: D799510-0100; spectrophotometrically at 400 nm); soil β-xylosidase activity was determined via the Assay Kit of Soil β-xylosidase Activity (No.: D799556-0100; spectrophotometrically at 400 nm); soil FDA hydrolase activity was determined via the Assay Kit of Soil FDA Hydrolase Activity (No.: D799552-0100; spectrophotometrically at 490 nm); soil sucrase activity was determined via the Assay Kit of Soil Sucrase Activity (No.: D799504-0100; spectrophotometrically at 540 nm); soil protease activity was determined via the Assay Kit of Soil Protease Activity (No.: D799540-0100; spectrophotometrically at 680 nm); soil urease activity was determined via the Assay Kit of Soil Urease Activity (No.: D799506-0100; spectrophotometrically at 630 nm) (Sangon Biotech Co., Ltd., Shanghai, China) with micromethod.

### 2.4. Determination of SBC’s Structure

To ascertain SBC’s structure, each soil sample was subjected to individual processing. The total DNA from all soil samples was extracted using the HiPure Soil DNA Kits (Magen, Guangzhou, China). The integrity of the total DNA was ascertained through electrophoresis on 1.2% (weight–volume) agarose gels. The quantity of DNA was determined by using the ABI StepOnePlus Real-Time PCR System (Life Technologies, Foster City, CA, USA).

The amplification of the V3–V4 region of the 16S rRNA genes of SBC was conducted via the universal primers (341F and 806R) as previously described [57,58]. The amplicons were extracted from 2% (weight–volume) agarose gels and purified by using the AxyPrep DNA Gel Extraction Kit (Axygen Biosciences, Union City, CA, USA) in accordance with the manufacturer’s instructions and then quantified by using the ABI StepOnePlus Real-Time PCR System (Life Technologies, Foster City, CA, USA). The purified amplicons were pooled in equimolar amounts and subjected to paired-end sequencing on the Illumina platform at GENE DENOVO Co., Ltd., Guangzhou, Guangdong, China, in accordance with the standard protocols.

The raw data containing adapters or low-quality reads would affect the following assembly and analysis. Thus, to obtain high-quality clean reads, the raw reads were further filtered according to the following rules by using FASTP v0.18.0: (1) Removing reads containing more than 10% of unknown nucleotides (N); (2) Removing reads containing less than 50% of bases with a quality (Q-value) > 20. The paired-end clean reads were merged as raw tags using FLASH v1.2.11 with a minimum overlap of 10 bp and mismatch error rates of 2%. The noisy sequences of the raw tags were filtered under specific filtering conditions to obtain high-quality, clean tags. The filtering conditions are as follows: (1) The break raw tags from the first low-quality base site where the number of bases in the continuous low-quality value (the default quality threshold is ≤3) reaches the set length (the default length is 3 bp); (2) The filter tags whose continuous high-quality base length is less than 75% of the tag length.

The clean tags were clustered into operational taxonomic units (OTUs) of ≥97% similarity using the UPARSE v9.2.64 pipeline. All chimeric tags were removed using the UCHIME algorithm, and we finally obtained effective tags for further analysis. The tag sequence with the highest abundance was selected as a representative sequence within each cluster. The representative OTU sequences or ASV sequences were classified into organisms by a naïve Bayesian model using the RDP classifier v2.2 based on the SILVA database v138.1, with a confidence threshold value of 0.8.

The abundance statistics of each taxonomy were visualized using Krona v2.6. The stacked bar plot of the community composition was visualized in the R project ggplot2 package v2.2.1. The circular layout representations of species abundance were graphed using Circos v0.69-3. The heatmap of species abundance was plotted by using the p-heatmap package v1.0.12 in the R project. The Pearson correlation analysis of species was calculated in the R project psych package v1.8.4. The OTU’s species index, Shannon’s diversity index, Simpson’s dominance index, Pielou’s evenness index, Chao1’s richness index, and ACE’s richness index were calculated in QIIME v1.9.1. The phylogenetic diversity index was calculated in Picante v1.8.2.

The sequence alignment was performed using Muscle v3.8.31, and the phylogenetic tree was constructed using FastTree v2.1, then weighted and unweighted unifrac distance matrices were generated using GuniFrac package v1.0 in the R project. Jaccard and Bray–Curtis distance matrix calculated in the R project Vegan package v2.5.3. The biomarker features in each group were screened using the LEfSe software v1.0. The multivariate statistical techniques, including PCoA (principal coordinates analysis) and NMDS (non-metric multi-dimensional scaling) of (Un) weighted unifrac, jaccard, and Bray–Curtis distances, were generated in the R project Vegan package v2.5.3 and plotted in the R project ggplot2 package v2.2.1.

### 2.5. Statistical Analysis

The normality and homogeneity of the determined variances were evaluated through the application of Shapiro–Wilk’s test and Bartlett’s test, respectively. A multiple comparison test was employed to analyze the statistical differences in the values of the physicochemical properties, C and N contents, enzymatic activities in soil, and SBC’s alpha diversity among the different treatments via the Tukey test. A two-way ANOVA was employed to assess the impacts of planted type and N form on the physicochemical properties, C and N contents, enzymatic activities in soil, and SBC’s alpha diversity. The effect size of each factor was also evaluated by using the Partial Eta Squared (*η*^2^) values, which were designed to inform the two-way ANOVA. Correlation patterns of soil physicochemical properties, soil C and N contents, soil enzymatic activities, and SBC’s alpha diversity were estimated using correlation analysis and principal component analysis (PCA). The threshold for statistical significance was set at *p* ≤ 0.05. Statistical analyses were conducted using IBM SPSS Statistics 26.0 (IBM, Inc., Armonk, NY, USA).

## 3. Results

### 3.1. The Influences of Planted Type

#### 3.1.1. Soil Physicochemical Properties

**Figure 1:**
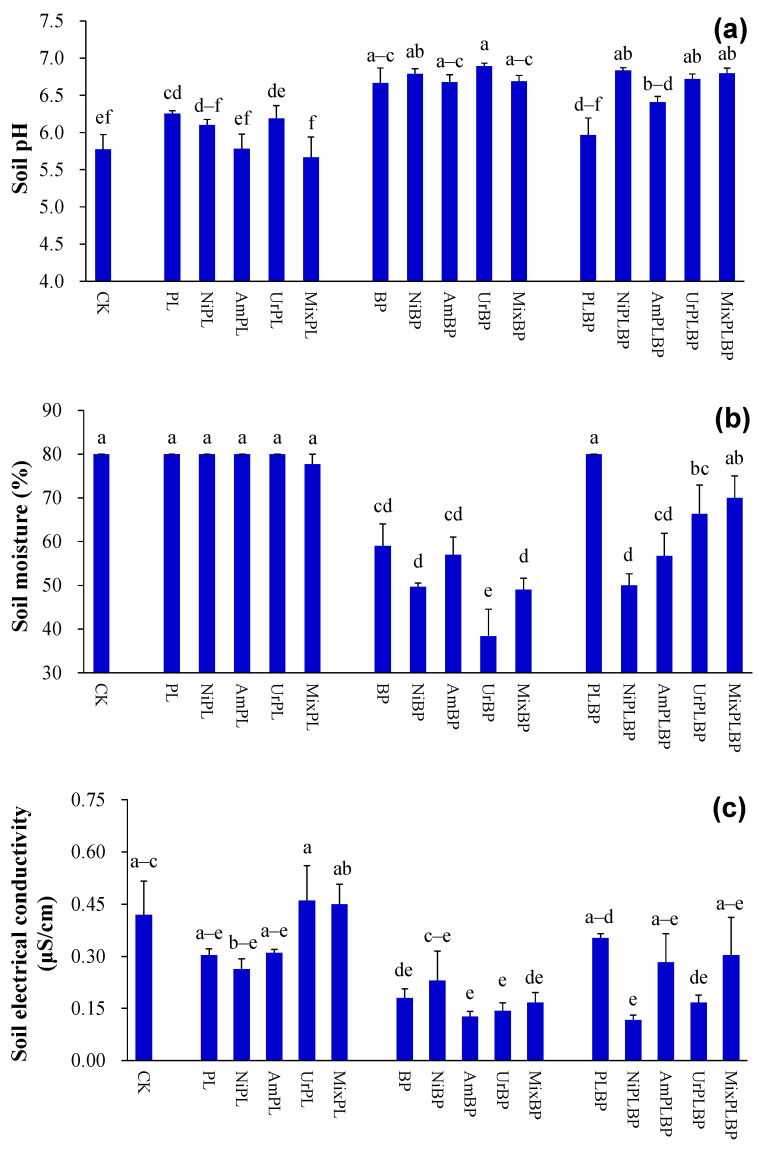
Soil physicochemical properties ((**a**) soil pH; (**b**) soil moisture; (**c**) soil electrical conductivity). Data (means ± SE; *n* = 3) with different lowercase letters indicate significant differences at 0.05 probability. Abbreviations: CK, control; Ni, nitrate; Am, ammonium; Ur, urea; Mix, mixed N; PL, monocultural *P. laciniata*; BP, monocultural *B. pilosa*; PLBP, co-cultivated *B. pilosa* and *P. laciniata*.

The soil pH under PL and BP was higher than that under the control (*p* < 0.05; Figure 1a). Soil pH under BP was also greater than that under BPPL (*p* < 0.05; Figure 1a). Soil pH under BP and BPPL was larger than that under PL treated with all forms of N (*p* < 0.05; Figure 1a).

Soil moisture under BP was less than that under the control, PL, and BPPL (*p* < 0.05; Figure 1b). Soil moisture under BP and BPPL was less than that under PL treated with nitrate and ammonium (*p* < 0.05; Figure 1b). Soil moisture under BP was lower than that under PL and BPPL treated with urea and mixed N (*p* < 0.05; Figure 1b).

Soil electrical conductivity under BP was lower than that under the control (*p* < 0.05; Figure 1c). Soil electrical conductivity under BP and BPPL was lower than that under PL treated with urea (*p* < 0.05; Figure 1c). Soil electrical conductivity under BP was less than that under PL treated with mixed N (*p* < 0.05; Figure 1c).

According to the two-way ANOVA analysis, planted type significantly affected soil pH, soil moisture, and soil electrical conductivity (*p* < 0.001; Table S1).

#### 3.1.2. Soil C and N Contents

**Figure 2:**
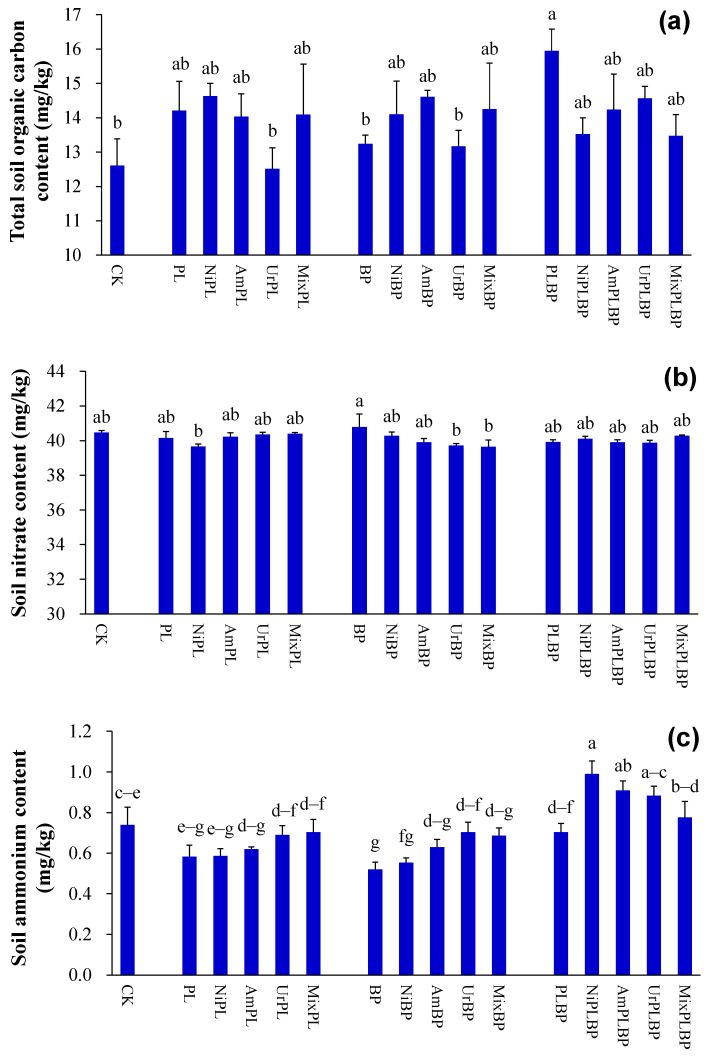
Soil carbon and nitrogen contents ((**a**) total soil organic carbon content; (**b**) soil nitrate content; (**c**) soil ammonium content). Data (means ± SE; *n* = 3) with different lowercase letters indicate significant differences at 0.05 probability. Data (i.e., the contents of soil microbial carbon, total soil organic nitrogen, and soil microbial nitrogen) without significant differences (*p* > 0.05) among the different treatments were not shown in this figure. Abbreviations: CK, control; Ni, nitrate; Am, ammonium; Ur, urea; Mix, mixed N; PL, monocultural *P. laciniata*; BP, monocultural *B. pilosa*; PLBP, co-cultivated *B. pilosa* and *P. laciniata*.

Total soil organic C content under BPPL was higher than that under the control and BP (*p* < 0.05; Figure 2a).

Soil ammonium content under BP was lower than that under the control and BPPL (*p* < 0.05; Figure 2b). Soil ammonium content under BPPL was higher than that under PL and BP treated with nitrate, ammonium, and urea (*p* < 0.05; Figure 2b).

According to the two-way ANOVA analysis, planted type significantly affected soil ammonium content (*p* < 0.001; Table S1).

#### 3.1.3. Soil Enzymatic Activities

**Figure 3:**
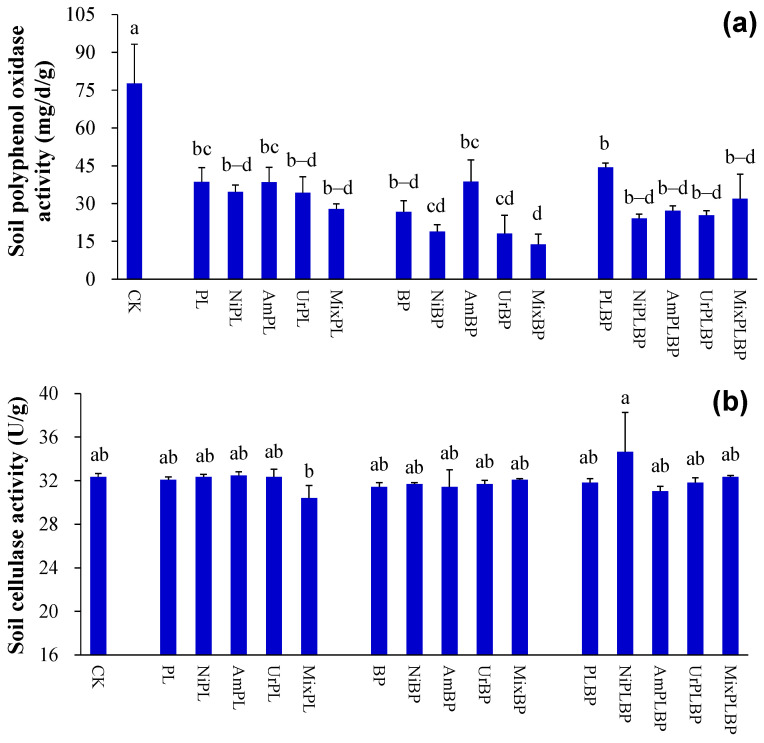

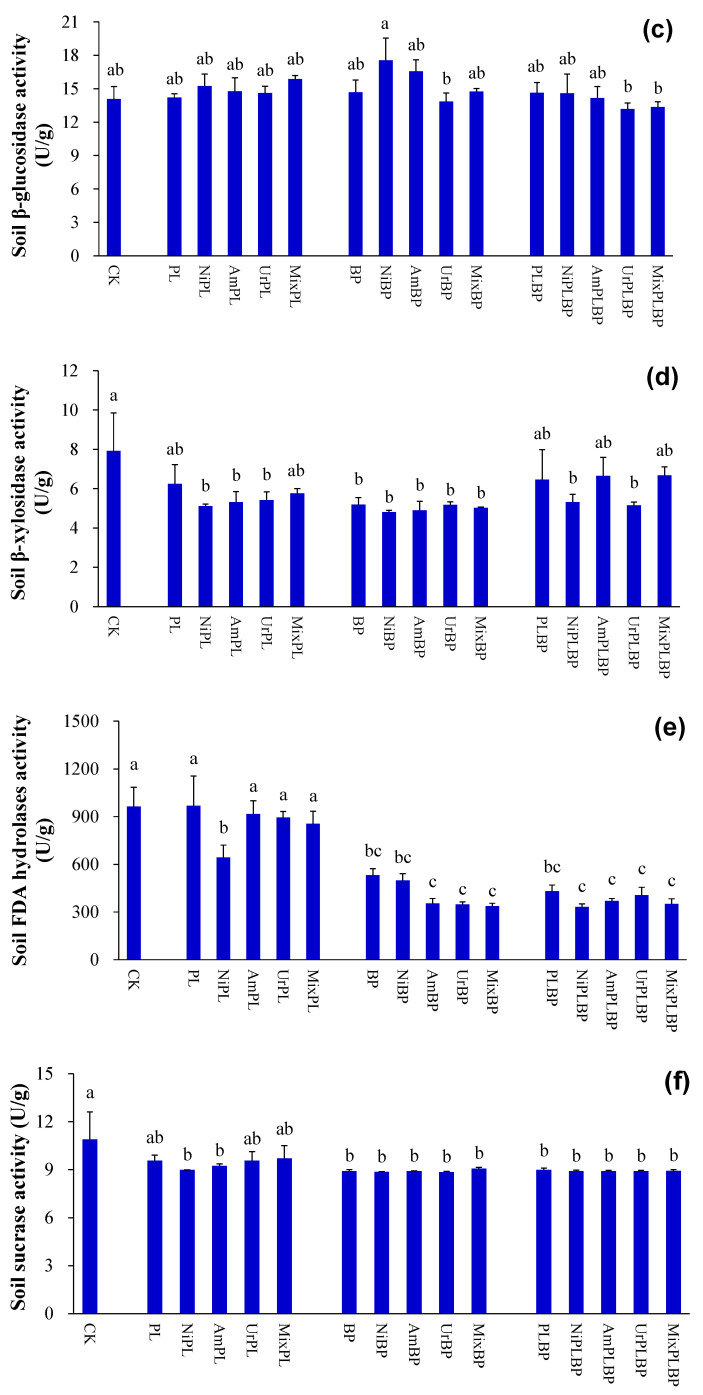

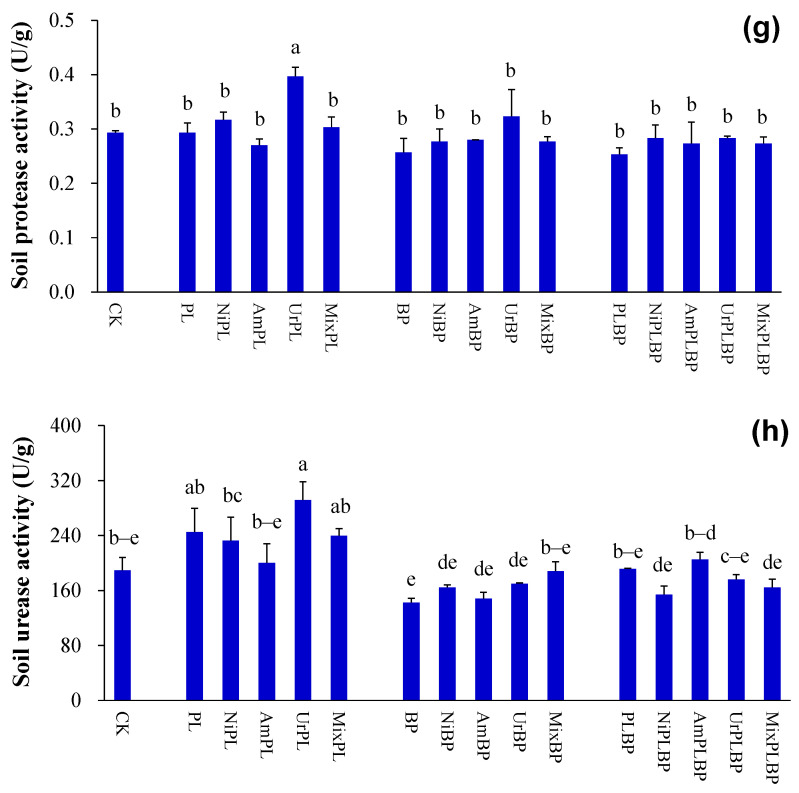
Soil enzymatic activities ((**a**) soil polyphenol oxidase activity; (**b**) soil cellulase activity; (**c**) soil β-glucosidase activity; (**d**) soil β-xylosidase activity; (**e**) soil FDA hydrolase activity; (**f**) soil sucrase activity; (**g**) soil protease activity; (**h**) soil urease activity). Data (means ± SE; *n* = 3) with different lowercase letters indicate significant differences at 0.05 probability. Abbreviations: CK, control; Ni, nitrate; Am, ammonium; Ur, urea; Mix, mixed N; PL, monocultural *P. laciniata*; BP, monocultural *B. pilosa*; PLBP, co-cultivated *B. pilosa* and *P. laciniata*.

Soil polyphenol oxidase activity under PL, BP, and BPPL was less than that under the control (*p* < 0.05; Figure 3a).

Soil β-xylosidase activity under BP was lower than that under the control (*p* < 0.05; Figure 3d).

Soil FDA hydrolase activity under BP and BPPL was lower than that under the control and PL (*p* < 0.05; Figure 3e). Soil FDA hydrolase activity under BPPL was less than that under PL treated with nitrate (*p* < 0.05; Figure 3e). Soil FDA hydrolase activity under BP and BPPL was less than that under PL treated with ammonium, urea, and mixed N (*p* < 0.05; Figure 3e).

Soil sucrase activity under BP and BPPL was less than that under the control (*p* < 0.05; Figure 3f).

Soil protease activity under BP and BPPL was lower than that under PL treated with urea (*p* < 0.05; Figure 3g).

Soil urease activity under BP was lower than that under PL (*p* < 0.05; Figure 3h). Soil urease activity under BP and BPPL was lower than that under PL treated with nitrate and urea (*p* < 0.05; Figure 3h). Soil urease activity under BPPL was lower than that under PL treated with mixed N (*p* < 0.05; Figure 3h).

According to the two-way ANOVA analysis, planted type significantly affected polyphenol oxidase activity, β-xylosidase activity, FDA hydrolase activity, sucrase activity, protease activity, and urease activity in soil (*p* < 0.05; Table S1).

#### 3.1.4. SBC’s Alpha Diversity

**Figure 4:**
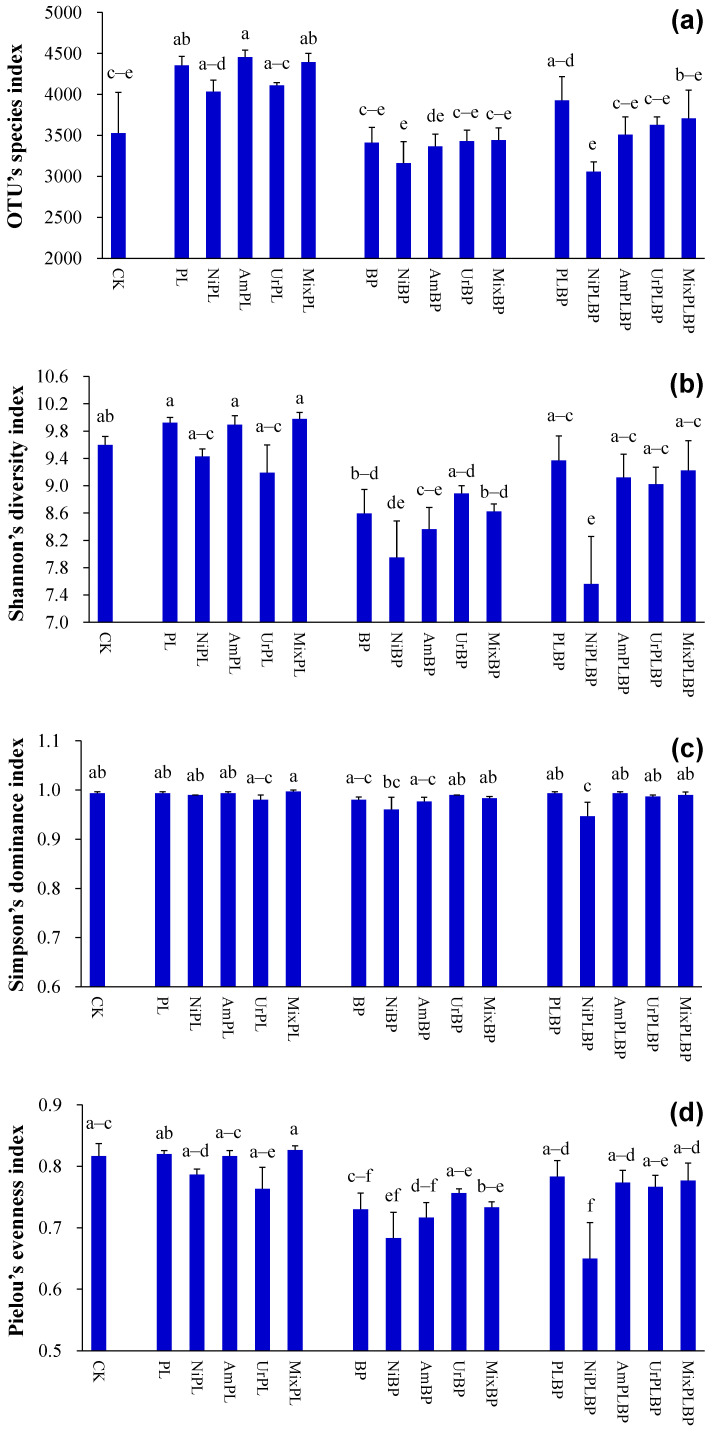

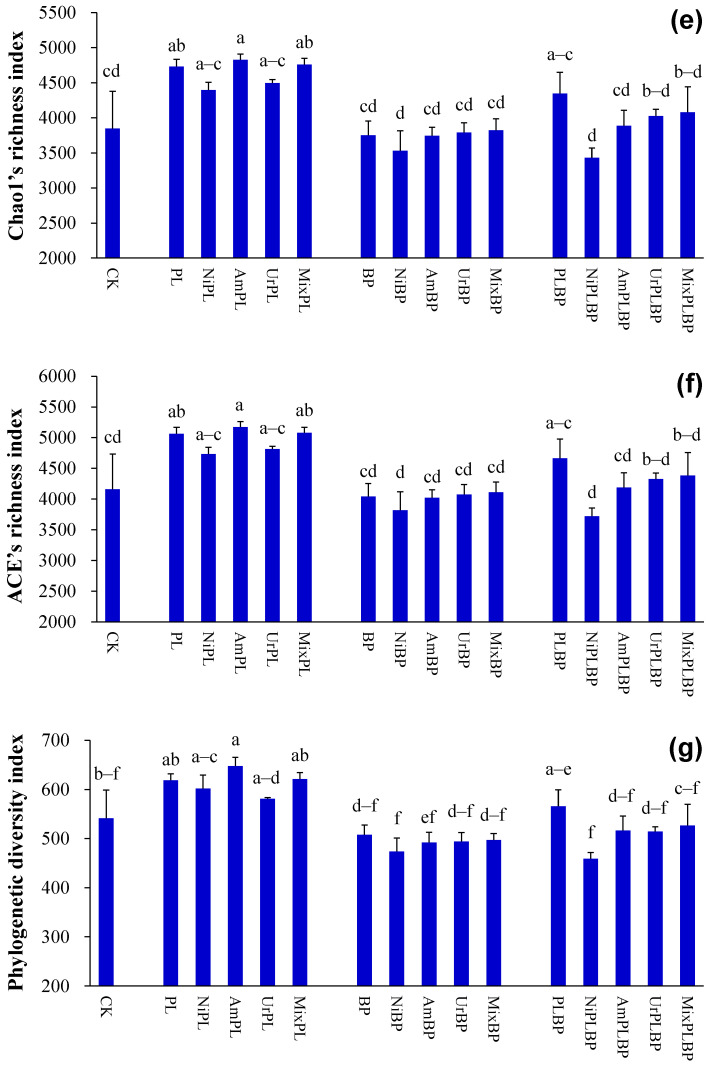
SBC’s alpha diversity ((**a**) OTU’s species index; (**b**) Shannon’s diversity index; (**c**) Simpson’s dominance index; (**d**) Pielou’s evenness index; (**e**) Chao1’s richness index; (**f**) ACE’s richness index; (**g**) phylogenetic diversity index). Data (means ± SE; *n* = 3) with different lowercase letters indicate significant differences at 0.05 probability. Abbreviations: CK, control; Ni, nitrate; Am, ammonium; Ur, urea; Mix, mixed N; PL, monocultural *P. laciniata*; BP, monocultural *B. pilosa*; PLBP, co-cultivated *B. pilosa* and *P. laciniata*.

The OTU’s species index under PL was larger than that under the control and BP (*p* < 0.05; Figure 4a). The OTU’s species index under BP and BPPL was less than that under PL treated with nitrate and ammonium (*p* < 0.05; Figure 4a). The OTU’s species index under BP was less than that under PL treated with mixed N (*p* < 0.05; Figure 4a).

The Shannon’s diversity index under BP was less than that under PL (*p* < 0.05; Figure 4b). The Shannon’s diversity index under BP was also lower than that under PL treated with nitrate, ammonium, and mixed N (*p* < 0.05; Figure 4b).

The Simpson’s dominance index under BPPL was less than that under PL treated with nitrate (*p* < 0.05; Figure 4c).

The Pielou’s evenness index under BP was less than that under PL (*p* < 0.05; Figure 4d). The Pielou’s evenness index under BP and BPPL was less than that under PL treated with nitrate (*p* < 0.05; Figure 4d). The Pielou’s evenness index under BP was less than that under PL treated with ammonium and mixed N (*p* < 0.05; Figure 4d).

The Chao1’s richness index and ACE’s richness index under PL were larger than those under the control and BP (*p* < 0.05; Figure 4e,f). The Chao1’s richness index and ACE’s richness index under BP and BPPL were lower than those under PL treated with nitrate and ammonium (*p* < 0.05; Figure 4e,f). The Chao1’s richness index and ACE’s richness index under BP were lower than those under PL treated with mixed N (*p* < 0.05; Figure 4e,f).

The phylogenetic diversity index under BP was lower than that under PL (*p* < 0.05; Figure 4g). The phylogenetic diversity index under BP and BPPL was lower than that under PL treated with all forms of N (*p* < 0.05; Figure 4g).

According to the results of the two-way ANOVA analysis, planted type significantly affected all SBC’s alpha diversity indices (except the Simpson’s dominance index) (*p* < 0.05; Table S1).

### 3.2. The Influences of N Form

#### 3.2.1. Soil Physicochemical Properties

Soil pH under PL treated with ammonium and mixed N was less than that under PL (*p* < 0.05; Figure 1a). Soil pH under PL treated with mixed N was also less than that under PL treated with urea (*p* < 0.05; Figure 1a). The soil pH under BPPL treated with nitrate, urea, and mixed N was greater than that under BPPL (*p* < 0.05; Figure 1a).

Soil moisture under BP treated with urea was less than that under BP and BP treated with nitrate, ammonium, and mixed N (*p* < 0.05; Figure 1b). Soil moisture under BPPL treated with nitrate, ammonium, and urea was lower than that under BPPL (*p* < 0.05; Figure 1b). Soil moisture under BPPL treated with nitrate and ammonium was also lower than that under BPPL treated with mixed N (*p* < 0.05; Figure 1b). Similarly, soil moisture under BPPL treated with nitrate was less than that under BPPL treated with urea (*p* < 0.05; Figure 1b).

Soil electrical conductivity under BP treated with urea was greater than that under BP treated with nitrate (*p* < 0.05; Figure 1c).

According to the two-way ANOVA analysis, N form significantly impacted soil pH and moisture (*p* < 0.05; Table S1).

#### 3.2.2. Soil C and N Contents

Soil nitrate content under BP treated with urea and mixed N was lower than that under BP (*p* < 0.05; Figure 2b).

Soil ammonium content under BP treated with urea was greater than that under BP (*p* < 0.05; Figure 2c). Soil ammonium content under BPPL treated with nitrate, ammonium, and urea was larger than that under BPPL (*p* < 0.05; Figure 2c). Soil ammonium content under BPPL treated with nitrate was also larger than that under BPPL treated with mixed N (*p* < 0.05; Figure 2c).

Based on the results of the two-way ANOVA analysis, N form significantly affected soil ammonium content (*p* < 0.01; Table S1).

#### 3.2.3. Soil Enzymatic Activities

Soil polyphenol oxidase activity under BP treated with ammonium was larger than that under BP treated with mixed N (*p* < 0.05; Figure 3a). Soil β-glucosidase activity under BP treated with nitrate was larger than that under BP treated with urea (*p* < 0.05; Figure 3c).

Soil FDA hydrolase activity under PL treated with nitrate was less than that under PL and PL treated with ammonium, urea, and mixed N (*p* < 0.05; Figure 3e).

Soil protease activity under PL treated with urea was lower than that under PL and PL treated with nitrate, ammonium, and mixed N (*p* < 0.05; Figure 3g).

Soil urease activity under PL treated with urea was larger than that under PL treated with nitrate and ammonium (*p* < 0.05; Figure 3h).

According to the two-way ANOVA analysis, N form significantly impacted soil protease activity (*p* < 0.01; Table S1).

#### 3.2.4. SBC’s Alpha Diversity

The OTU’s species index and Simpson’s dominance index under BPPL treated with nitrate were lower than those under BPPL (*p* < 0.05; Figure 4a,c).

The Shannon’s diversity index and Pielou’s evenness index under BPPL treated with nitrate were lower than those under BPPL and BPPL treated with ammonium, urea, and mixed N (*p* < 0.05; Figure 4b,d).

The Chao1’s richness index, ACE’s richness index, and phylogenetic diversity index under BPPL treated with nitrate were less than those under BPPL (*p* < 0.05; Figure 4e–g).

According to the two-way ANOVA analysis, N form significantly affected Simpson’s dominance index, Pielou’s evenness index, and Chao1’s richness index (*p* < 0.01; Table S1).

### 3.3. Correlation Patterns of the Physicochemical Properties, C and N Contents, and Enzymatic Activities in Soil, and SBC’s Alpha Diversity

According to the correlation analysis, soil pH and soil microbial C content were significantly negatively correlated with all indices of SBC’s alpha diversity (*p* < 0.05; Table S2). Similarly, soil ammonium content was significantly negatively correlated with the phylogenetic diversity index (*p* < 0.05; Table S2). Soil moisture and soil electrical conductivity were significantly positively correlated with all indices of SBC’s alpha diversity (*p* < 0.05; Table S2). In addition, soil FDA hydrolase activity and soil urease activity were significantly positively correlated with all indices of SBC’s alpha diversity except Simpson’s dominance index (*p* < 0.05; Table S2). Soil β-xylosidase activity and soil sucrase activity were also significantly positively correlated with Pielou’s evenness index (*p* < 0.05; Table S2).

**Figure 5:**
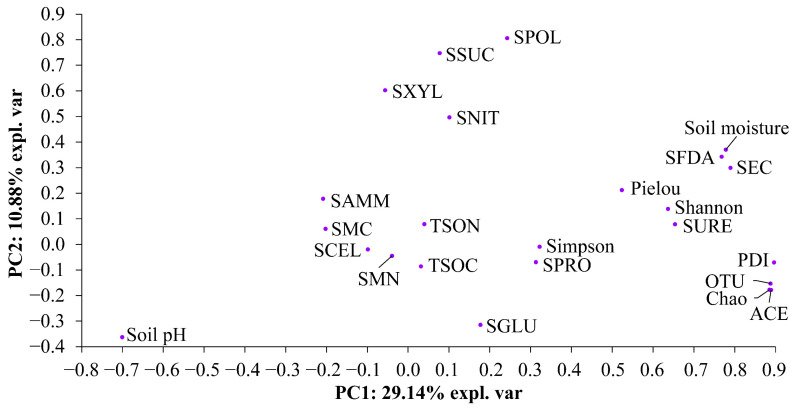
PCA of correlation patterns of the physicochemical properties, C and N contents, enzymatic activities in soil, and SBC’s alpha diversity. The *X*-axis and *Y*-axis account for 29.14% and 10.88% of the total variation, respectively. Abbreviations: SEC, soil electrical conductivity; TSOC, total soil organic carbon content; SMC, soil microbial carbon content; TSON, total soil organic nitrogen content; SMN, soil microbial nitrogen content; SNIT, soil nitrate content; SAMM, soil ammonium content; SPOL, soil polyphenol oxidase activity; SCEL, soil cellulase activity; SGLU, soil β-glucosidase activity; SXYL, soil β-xylosidase activity; SFDA, soil FDA hydrolase activity; SSUC, soil sucrase activity; SPRO, soil protease activity; SURE, soil urease activity; OTU, OTU’s species index; Shannon, Shannon’s diversity index; Simpson, Simpson’s dominance index; Pielou, Pielou’s evenness index; Chao, Chao1’s richness index; ACE, ACE’s richness index; PDI, phylogenetic diversity index.

Strong correlations were observed between SBC’s alpha diversity and moisture, electrical conductivity, FDA hydrolase activity, protease activity, and urease activity in soil (Figure 5).

### 3.4. SBC’s Structure

The mean of the Good’s coverage index across all samples was ~0.9772. The SBC’s beta diversity treated with different planted types and N forms presented noticeable differences according to the weighted UniFrac distances (Figures S2–S4).

The leading role of the principal SBC’s biomarkers, Parcubacteria, Anaerolineae, and Saccharimonadia, was notably improved under PL compared to the control. The leading role of the principal SBC’s biomarkers, Blastocatellia, Saccharimonadia, and Cyanobacteriia, was notably improved under PL treated with nitrate compared to PL. The leading role of the principal SBC’s biomarkers, Chloroflexia and Saccharimonadia, was notably improved under PL treated with ammonium compared to PL. The leading role of the principal SBC’s biomarkers, Parcubacteria, Gammaproteobacteria, Saccharimonadia, and Bacteroidia, was notably improved under PL treated with urea compared to PL. The leading role of the principal SBC’s biomarkers, Parcubacteria and Saccharimonadia, was notably improved under PL treated with mixed N compared to PL (Figure S5a).

The leading role of the principal SBC’s biomarkers, Blastocatellia, Saccharimonadia, Cyanobacteriia, and Alphaproteobacteria, was notably improved under BP compared to the control. The leading role of the principal SBC’s biomarker, Saccharimonadia, was notably improved under BP treated with all forms of N compared to BP (Figure S5b).

The leading role of the principal SBC’s biomarkers, Parcubacteria and Saccharimonadia, was notably improved under BPPL compared to the control. The leading role of the principal SBC’s biomarkers, Chloroflexia and Saccharimonadia, was notably improved under BPPL treated with nitrate compared to BPPL. The leading role of the principal SBC’s biomarker, Saccharimonadia, was notably improved under BPPL treated with ammonium, urea, and mixed N compared to BPPL (Figure S5c).

Based on the results of the LEfSe analyses, numerous SBC’s taxa (i.e., leading biomarkers) were principally altered when treated with different planted types and N forms (Figure S6).

## 4. Discussion

The results of this study demonstrated that BP resulted in a notable increase in soil pH in comparison to the control. As a result, the soil undergoes alkalization rather than acidification as a consequence of the *B. pilosa* invasion. This finding is likely attributable to the exudation of alkaline substances or other substances that can trigger the alkaline effect, such as anions, during the growth of *B. pilosa* [59,60,61,62]. Furthermore, this phenomenon may be attributed to a decrease in the exudation of hydrogen ions via a decrease in the release of acid-containing substances present in the root exudates of *B. pilosa* [8,63,64,65]. Additionally, the alkalization of soil under *B. pilosa* invasion may be attributed to the selective absorption of different forms of N components in soil by *B. pilosa*, a phenomenon that has been observed in numerous other IPs [66,67,68,69]. In particular, BP was observed to significantly decrease soil ammonium content in comparison to the control. Consequently, *B. pilosa* exhibits a preference for ammonium absorption over other nitrogenous components, a trait that has also been observed in other plants (including other IPS) [40,70,71]. This finding may indicate that the uptake capacity and utilization efficiency of ammonium by *B. pilosa* may be greater than those of other forms of N components, primarily due to the lower energy cost associated with ammonium uptake and utilization [72,73].

BP exhibited a notable reduction in soil moisture in comparison to the control. This finding may be attributed to the rapid evapotranspiration rate of IPS, which is mediated by their rapid growth pattern, especially high water-use efficiency [74,75,76,77].

A notable reduction in soil electrical conductivity was observed under BP in comparison to the control. This finding may be attributed to a reduction in the exudation of secondary compounds, especially root exudates, of *B. pilosa*, which has resulted in a decrease in soil water-soluble salt content. Prior research has indicated a strong correlation between the exudate profiles of plants and soil electrical conductivity [78,79,80,81].

BPPL resulted in a notable enhancement in total soil organic C content compared to the control. This phenomenon may be attributed to the increased content of soil C, which is the result of the contributions of C-containing substances from the two plants to the soil, leading to C sequestration in the soil [61,82,83,84]. Thus, soil organic C content can be increased under BPPL. It can, therefore, be concluded that there is a greater potential for organic C to be sequestered in soil, particularly through the utilization of C by soil microorganisms. The results of this study indicate that BPPL may serve as a vital C sink in soil rather than a C emitter through the process of C sequestration in soil. It can thus be argued that BPPL may contribute to the alleviation of climate change rather than its exacerbation. It can thus be proposed that, although IPs have a negative ecological impact in many cases, they can also be transformed into a valuable resource if managed properly, at least in terms of their impact on soil organic C content.

It has been demonstrated that the introduction of IPs can result in alterations to soil enzymatic activities [40,85,86] and SBC’s alpha diversity [87,88,89,90]. This is attributed to the release of nutrients (especially C/N-containing substances) through the decomposition of litter and/or the exudation of root substances during the growth process of the plants. Inconsistent with the first hypothesis, BP markedly reduced the activities of polyphenol oxidase, β-xylosidase, FDA hydrolase, and sucrase in the soil in comparison to the control. It can thus be concluded that the ability of polyphenols to oxidize, the ability of xylan degradation, the ability of FDA to hydrolyze, and the ability of sucrose to hydrolyze in soil decreased as a consequence of *B. pilosa* invasion. The observed decline in soil enzymatic activities may be attributed to a reduction in the nutrient availability level in the soil and/or an increase in SBC’s metabolic rate under *B. pilosa* invasion. Inconsistent with the first hypothesis, BP also exhibited a marked reduction in all SBC’s alpha diversity indices (with the exception of Simpson’s dominance index) in comparison to PL. Furthermore, previous studies have demonstrated that IPs can reduce SBC’s alpha diversity [87,88,89,90]. The reduction in SBC’s alpha diversity observed in the presence of *B. pilosa* invasion may be attributed to a number of issues, e.g., the elevated soil pH and the reduced moisture, electrical conductivity, ammonium content, and enzymatic activities. In particular, these indicators may have a significant impact on SBC’s diversity and abundance, primarily through the fluctuations in resource use and access to nutrients metabolized by SBC. Furthermore, the aforementioned indicators also demonstrated a robust correlation with SBC’s alpha diversity, as evidenced by the findings of the correlation analysis and PCA. Moreover, BP resulted in notable alterations to SBC’s beta diversity, with the appearance of several leading SBC’s biomarkers. This phenomenon may be attributed to the differences in the root exudates of the two plants, which have caused a certain degree of differentiation at the species level in SBC. In particular, the presence of several principal SBC’s biomarkers may be attributed to alterations in the form, amount, and complexity of nutrients (e.g., C/N-containing substances) in soil. These alterations result in selective facilitative or inhibitory effects on SBC. Consequently, some of SBC’s taxa are able to flourish, while others may become eradicated over time due to the encroachment of *B. pilosa*. As a result, the invasion of *B. pilosa* exerts a notable influence not only on SBC’s alpha diversity but also on SBC’s community composition.

The study demonstrated that soil FDA hydrolase activity and SBC’s alpha diversity, especially the indices of Shannon’s diversity, Simpson’s dominance, and Pielou’s evenness, exhibited a pronounced decline in response to nitrate treatment in BPPL treated with nitrate compared to those under BPPL when compared to those treated with ammonium, urea, and mixed N. This result is consistent with the second hypothesis. This finding can be attributed to the stronger reduction in soil moisture and electrical conductivity treated with nitrate compared to that treated with ammonium, urea, and mixed N. In particular, soil moisture [81,91,92,93] and electrical conductivity [81,94,95,96] are generally considered to be the main drivers of SBC’s diversity and abundance, mainly via the fluctuations in resource use and access to nutrients metabolized by SBC. Furthermore, the findings of the correlation analysis and PCA indicated a robust correlation between soil moisture, electrical conductivity, and SBC’s alpha diversity. In addition, all forms of N appeared to regulate several principal SBC’s biomarkers in either an up- or down-regulatory manner. This result may be attributed to the differing N-phototrophic capabilities of the principal SBC’s biomarkers. It can be postulated that the diverse forms of nitrogen may exert disparate selection pressures on the heterogeneous taxa of the principal SBC’s biomarkers, resulting in an expansion of N-phototrophic SBC taxa and a contraction of N-sensitive SBC taxa. It can be concluded that the N form primarily affects the composition of the soil bacterial community rather than its alpha diversity.

## 5. Conclusions

This study represents the inaugural attempt to clarify the effects of *B. pilosa* on soil physicochemical properties, soil C and N contents, soil enzymatic activities, and SBC’s community composition in comparison to *P. laciniata* treated with simulated N deposition at 5 g N m^−2^ yr^−1^ in four forms (nitrate, ammonium, urea, and mixed N). The primary findings are as follows: (1) BP caused a noteworthy elevation in soil pH, accompanied by a pronounced reduction in the moisture, electrical conductivity, ammonium content, and the activities of polyphenol oxidase, β-xylosidase, FDA hydrolase, and sucrase in soil when compared to the control. (2) BPPL mediated a substantial reduction in total soil organic C content compared to the control. (3) BP recruited a marked decrease in all SBC’s alpha diversity indices (with the exception of the Simpson’s dominance index) compared to PL. (4) Soil FDA hydrolase activity and SBC’s alpha diversity, in particular the indices of Shannon’s diversity, Simpson’s dominance, and Pielou’s evenness, were found to be significantly lower in BPPL treated with nitrate than in BPPL treated with ammonium, urea, and mixed N.

## Data Availability

The raw data supporting the conclusions of this article will be made available by the authors on request.

## Supplementary Materials

The following supporting information can be downloaded at: https://www.mdpi.com/article/10.3390/microorganisms12081624/s1, Table S1: Two-way ANOVA on the influences of planted type and nitrogen form on soil physicochemical properties, soil carbon and nitrogen contents, soil enzymatic activities, and soil bacterial alpha diversity; Table S2: Correlations (*r*) between soil physicochemical properties, soil carbon and nitrogen contents, soil enzymatic activities, and soil bacterial alpha diversity; Figure S1: The geographical location (Zhenjiang, Jiangsu, China) of the sampled zone (square with red) in this study; Figure S2: PCoA of beta diversity estimates of soil bacterial communities based on the weighted UniFrac distance; Figure S3: NMDS of beta diversity estimates of soil bacterial communities based on the weighted UniFrac distance; Figure S4: Heatmap of beta diversity estimates of soil bacterial communities based on the weighted UniFrac distance at the family level. The color blocks represent the distance values; Figure S5: Bubble chart of soil bacterial biomarkers at the class level; Figure S6: LEfSe method identifies the significantly different abundant taxa of soil bacterial communities with different treatments.
