# Supplementary material for: Does Bidens pilosa L. Affect Carbon and Nitrogen Contents, Enzymatic Activities, and Bacterial Communities in Soil Treated with Different Forms of Nitrogen Deposition?
Source: Microorganisms. 2024 Aug 9;12(8):1624. doi: 10.3390/microorganisms12081624 (PMC11356382; doi:10.3390/microorganisms12081624)
Supplement: Supplementary file 1 [file microorganisms-12-01624-s001.zip › microorganisms-3129178-supplementary.pdf]

Electronic supplementary materials:

Table S1 Two-way ANOVA on the influences of planted type and nitrogen form on soil physicochemical properties, soil carbon and nitrogen contents, soil enzymatic activities, and soil bacterial alpha diversity.

Variable and source of variation		Type III Sum of Squares	df	Mean Square	*F*	*p*
Planted type	Soil pH	5.065	3	1.688	28.220	< **0.001*** **
	Soil moisture	0.639	3	0.213	57.902	< **0.001*** **
	Soil electrical conductivity	0.313	3	0.104	10.516	< **0.001*** **
	Total soil organic carbon content	9.888	3	3.296	1.810	0.165
	Soil microbial carbon content	150.021	3	50.007	0.704	0.556
	Total soil organic nitrogen content	7.021	3	2.340	1.276	0.299
	Soil microbial nitrogen content	38.206	3	12.735	1.300	0.291
	Soil nitrate content	0.232	3	0.077	0.357	0.784
	Soil ammonium content	0.551	3	0.184	23.564	< **0.001*** **
	Soil polyphenol oxidase activity	4824.668	3	1608.223	13.552	< **0.001*** **
	Soil cellulase activity	4.137	3	1.379	0.397	0.756
	Soil β-glucosidase activity	17.498	3	5.833	1.851	0.158
	Soil β-xylosidase activity	16.552	3	5.517	3.227	**0.035***
	Soil FDA hydrolase activity	2348452.810	3	782817.603	51.285	< **0.001*** **
	Soil sucrase activity	9.143	3	3.048	3.939	**0.017***
	Soil protease activity	0.017	3	0.006	3.967	**0.016***

	Soil urease activity	52994.268	3	17664.756	18.495	**< 0.001*** **
	OTU's species index	7105430.961	3	2368476.987	17.180	**< 0.001*** **
	Shannon's diversity index	11.512	3	3.837	11.782	**< 0.001*** **
	Simpson's dominance index	0.001	3	< 0.001	1.302	0.291
	Pielou's evenness index	0.052	3	0.017	8.563	**< 0.001*** **
	Chao1's richness index	7205298.347	3	2401766.116	15.850	**< 0.001*** **
	ACE's richness index	7857404.365	3	2619134.788	15.600	**< 0.001*** **
	Phylogenetic diversity index	124952.198	3	41650.733	20.349	**< 0.001*** **
Nitrogen form	Soil pH	0.809	4	0.202	3.382	**0.020***
	Soil moisture	0.092	4	0.023	6.266	**0.001****
	Soil electrical conductivity	0.055	4	0.014	1.385	0.261
	Total soil organic carbon content	5.792	4	1.448	0.795	0.537
	Soil microbial carbon content	182.137	4	45.534	0.641	0.637
	Total soil organic nitrogen content	0.091	4	0.023	0.012	1.000
	Soil microbial nitrogen content	4.179	4	1.045	0.107	0.979
	Soil nitrate content	0.542	4	0.135	0.626	0.647
	Soil ammonium content	0.126	4	0.031	4.035	**0.009****
	Soil polyphenol oxidase activity	1155.735	4	288.934	2.435	0.067
	Soil cellulase activity	10.161	4	2.540	0.731	0.578
	Soil β-glucosidase activity	18.600	4	4.650	1.475	0.233
	Soil β-xylosidase activity	5.068	4	1.267	0.741	0.571
	Soil FDA hydrolase activity	121490.983	4	30372.746	1.990	0.120

	Soil sucrase activity	0.561	4	0.140	0.181	0.946
	Soil protease activity	0.025	4	0.006	4.438	**0.006****
	Soil urease activity	4927.263	4	1231.816	1.290	0.295
	OTU’s species index	1274033.200	4	318508.300	2.310	0.079
	Shannon’s diversity index	1274033.200	4	318508.300	2.310	0.079
	Simpson’s dominance index	5.864	4	1.466	4.501	**0.005****
	Pielou’s evenness index	0.004	4	0.001	2.807	**0.042***
	Chao1’s richness index	0.032	4	0.008	4.028	**0.009****
	ACE’s richness index	1314664.696	4	328666.174	2.169	0.095
	Phylogenetic diversity index	1346236.183	4	336559.046	2.005	0.118
Planted type × Nitrogen form	Soil pH	1.701	8	0.213	3.554	**0.005****
	Soil moisture	0.152	8	0.019	5.176	**< 0.001*****
	Soil electrical conductivity	0.181	8	0.023	2.276	**0.047***
	Total soil organic carbon content	19.099	8	2.387	1.311	0.273
	Soil microbial carbon content	407.539	8	50.942	0.718	0.675
	Total soil organic nitrogen content	2.734	8	0.342	0.186	0.991
	Soil microbial nitrogen content	17.502	8	2.188	0.223	0.984
	Soil nitrate content	3.487	8	0.436	2.014	0.077
	Soil ammonium content	0.144	8	0.018	2.310	**0.044***
	Soil polyphenol oxidase activity	1047.272	8	130.909	1.103	0.387
	Soil cellulase activity	22.316	8	2.790	0.802	0.605
	Soil β-glucosidase activity	19.783	8	2.473	0.785	0.619

Soil β-xylosidase activity	4.440	8	0.555	0.325	0.950
Soil FDA hydrolase activity	192240.371	8	24030.046	1.574	0.172
Soil sucrase activity	0.604	8	0.076	0.098	0.999
Soil protease activity	0.012	8	0.002	1.097	0.390
Soil urease activity	16891.912	8	2111.489	2.211	0.053
OTU's species index	546179.067	8	68272.383	0.495	0.850
Shannon's diversity index	546179.067	8	68272.383	0.495	0.850
Simpson's dominance index	3.620	8	0.452	1.389	0.239
Pielou's evenness index	0.003	8	< 0.001	1.137	0.366
Chao1's richness index	0.023	8	0.003	1.425	0.224
ACE's richness index	611343.239	8	76417.905	0.504	0.844
Phylogenetic diversity index	668046.991	8	83505.874	0.497	0.849

The symbols *, **, and *** indicate significant differences at the 0.05, 0.01, and 0.001 probability level, respectively. *p* values equal to or less than 0.05 are shown in bold.

Table S2 Correlations (r) between soil physicochemical properties, soil carbon and nitrogen contents, soil enzymatic activities, and soil bacterial alpha diversity (n = 3).

		OTU's species index	Shannon's diversity index	Simpson's dominance index	Pielou's evenness index	Chao1's richness index	ACE's richness index	Phylogenetic diversity index
Soil pH	r	-0.559^{***}	-0.534^{***}	-0.318^{*}	-0.489^{***}	-0.552^{***}	-0.556^{***}	-0.619^{***}
	p	**< 0.001**	**< 0.001**	**0.027**	**< 0.001**	**< 0.001**	**< 0.001**	**< 0.001**
Soil moisture	r	0.662^{***}	0.619^{***}	0.306^{*}	0.562^{***}	0.650^{***}	0.653^{***}	0.711^{***}
	p	**< 0.001**	**< 0.001**	**0.034**	**< 0.001**	**< 0.001**	**< 0.001**	**< 0.001**
Soil electrical	r	0.601^{***}	0.530^{***}	0.289^{*}	0.466^{**}	0.597^{***}	0.601^{***}	0.611^{***}
conductivity	p	**< 0.001**	**< 0.001**	**0.046**	**0.001**	**< 0.001**	**< 0.001**	**< 0.001**
Total soil organic	r	0.169	0.064	0.119	0.049	0.180	0.181	0.142
carbon content	p	0.250	0.668	0.419	0.739	0.220	0.219	0.336
Soil microbial carbon	r	-0.411^{**}	-0.378^{**}	-0.407^{**}	-0.357^{*}	-0.409^{**}	-0.403^{**}	-0.372^{**}
content	p	**0.004**	**0.008**	**0.004**	**0.013**	**0.004**	**0.005**	**0.009**
Total soil organic	r	0.059	-0.016	-0.155	-0.024	0.053	0.048	0.069
nitrogen content	p	0.688	0.916	0.292	0.870	0.723	0.748	0.642
Soil microbial	r	-0.046	0.071	0.193	0.088	-0.040	-0.036	-0.063
nitrogen content	p	0.757	0.630	0.190	0.553	0.785	0.806	0.673
Soil nitrate content	r	0.143	0.173	-0.004	0.172	0.130	0.128	0.148
	p	0.331	0.239	0.978	0.243	0.377	0.387	0.317
Soil ammonium	r	-0.284	-0.209	-0.170	-0.177	-0.274	-0.272	-0.317^{*}

content	*p*	0.050	0.154	0.248	0.229	0.060	0.062	**0.028**
Soil polyphenol	*r*	0.057	0.244	0.116	0.284	0.039	0.043	0.153
oxidase activity	*p*	0.698	0.095	0.433	0.050	0.794	0.773	0.301
Soil cellulase activity	*r*	-0.004	0.048	0.069	0.050	0.001	< 0.001	0.004
	p	0.978	0.746	0.641	0.734	0.996	0.998	0.976
Soil β-glucosidase	*r*	0.108	< 0.001	0.034	-0.028	0.107	0.104	0.134
activity	*p*	0.466	0.999	0.819	0.850	0.469	0.480	0.364
Soil β-xylosidase	*r*	-0.121	0.246	0.190	0.311*	-0.147	-0.149	-0.063
activity	*p*	0.412	0.092	0.196	**0.032**	0.320	0.313	0.673
Soil FDA hydrolase	*r*	0.625***	0.536***	0.241	0.478**	0.601***	0.603***	0.685***
activity	*p*	**< 0.001**	**< 0.001**	0.099	**0.001**	**< 0.001**	**< 0.001**	**< 0.001**
Soil sucrase activity	*r*	-0.066	0.219	0.049	0.292*	-0.091	-0.098	-0.004
	p	0.655	0.134	0.741	**0.044**	0.541	0.509	0.978
Soil protease activity	*r*	0.182	0.083	-0.011	0.055	0.179	0.177	0.157
	p	0.216	0.576	0.941	0.710	0.224	0.230	0.287
Soil urease activity	*r*	0.495***	0.454**	0.198	0.410**	0.488***	0.489***	0.498***
	p	**< 0.001**	**0.001**	0.178	**0.004**	**< 0.001**	**< 0.001**	**< 0.001**

The symbols *, **, and *** indicate significant differences at the 0.05, 0.01, and 0.001 probability level, respectively. *p* values equal to or less than 0.05 are shown in bold.

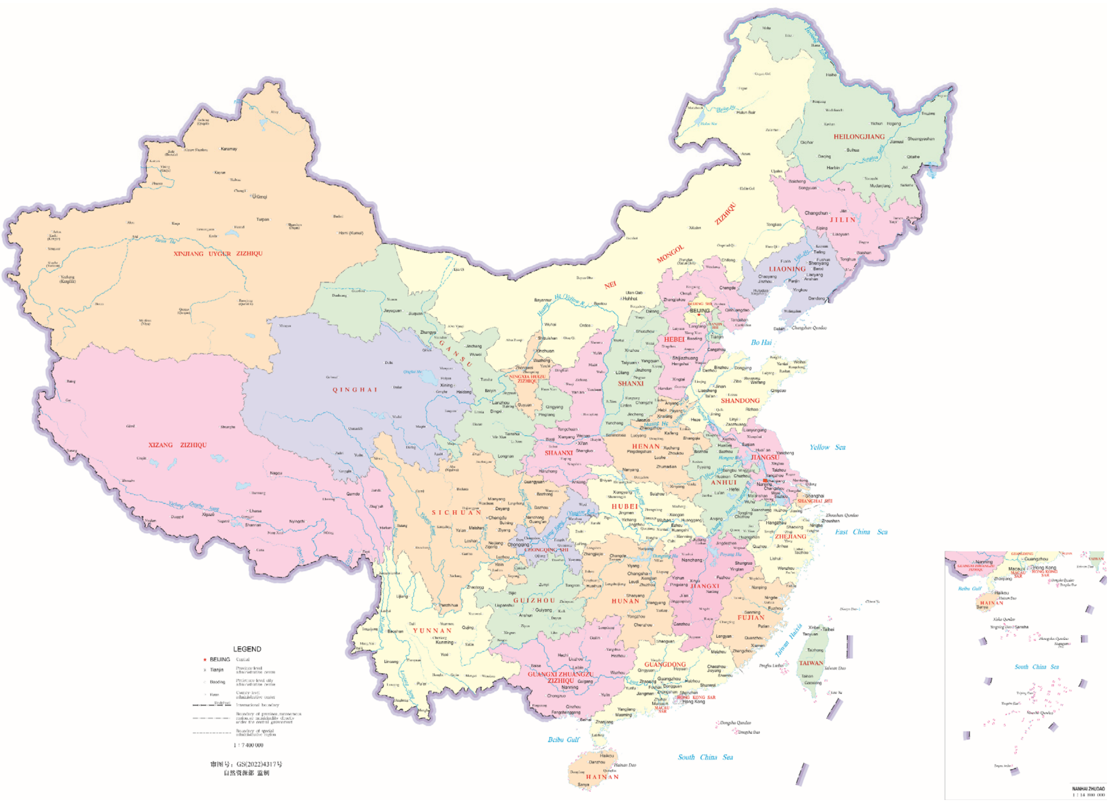

Figure S1 The geographical location (Zhenjiang, Jiangsu, China) of the sampled zone (square with red) in this study (Map number: GS(2022)4317; produced by the Ministry of Natural Resources of China; http://bzdt.ch.mnr.gov.cn; accessed on 23 June 2024).

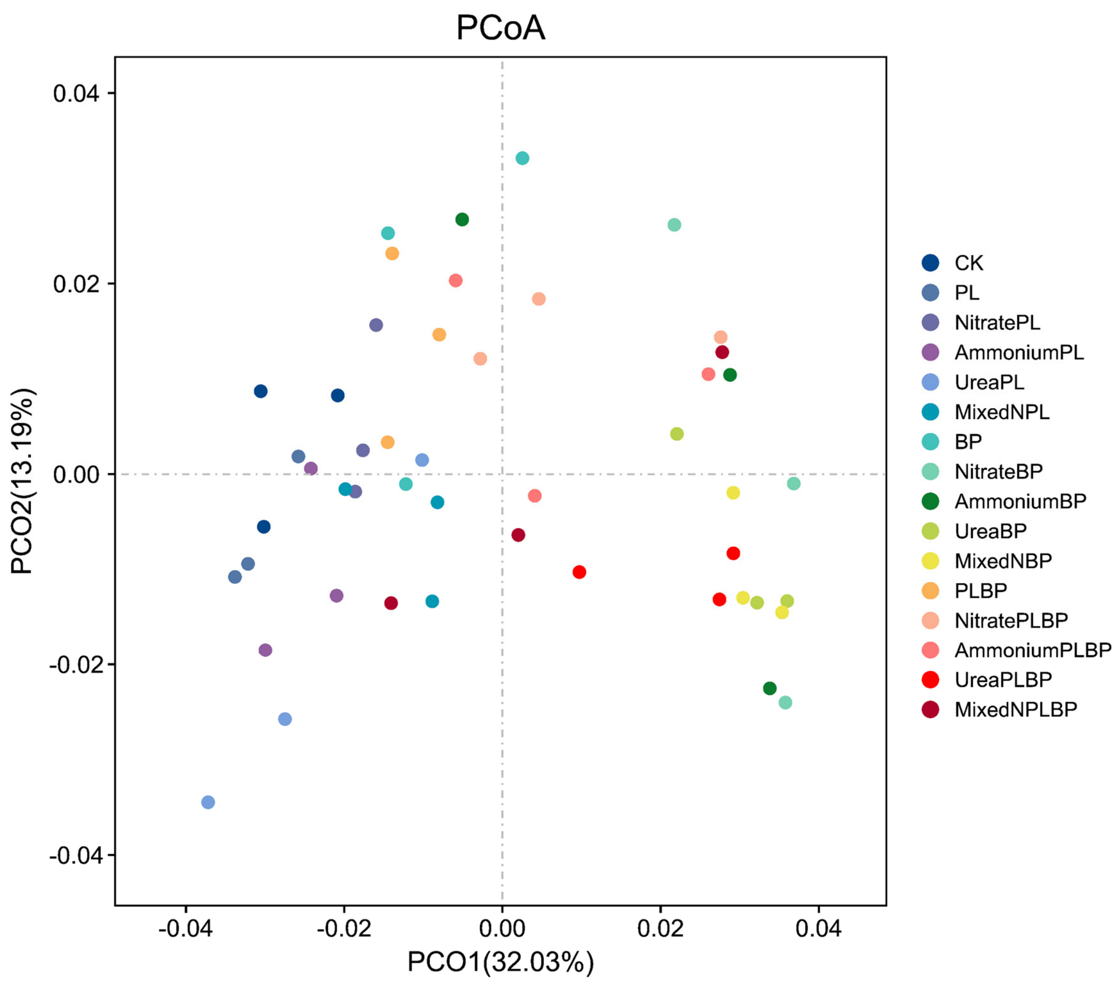

Figure S2 PCoA of beta diversity estimates of soil bacterial communities based on the weighted UniFrac distance. Abbreviations: CK, control; Ni, nitrate; Am, ammonium; Ur, urea; MixedN, mixed nitrogen; PL, monocultural *Pterocypsela laciniata* (Houtt.) Shih; BP, monocultural *Bidens pilosa* L.; PLBP, co-cultivated *Bidens pilosa* L. and *Pterocypsela laciniata* (Houtt.) Shih. The *X*-axis and *Y*-axis account for 32.03% and 13.19% of the total variation, respectively.

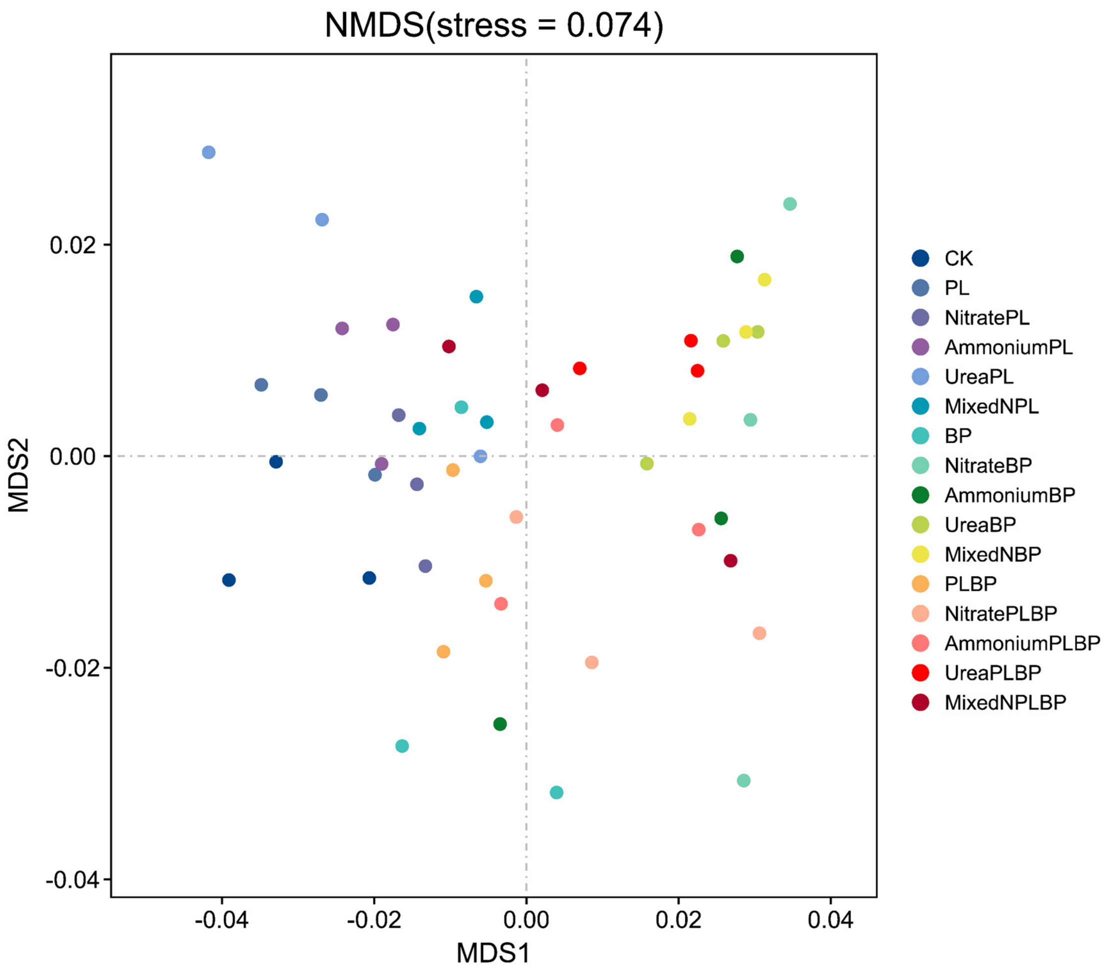

Figure S3 NMDS of beta diversity estimates of soil bacterial communities based on the weighted UniFrac distance. Abbreviations: CK, control; Ni, nitrate; Am, ammonium; Ur, urea; MixedN, mixed nitrogen; PL, monocultural *Pterocypsela laciniata* (Houtt.) Shih; BP, monocultural *Bidens pilosa* L.; PLBP, co-cultivated *Bidens pilosa* L. and *Pterocypsela laciniata* (Houtt.) Shih.

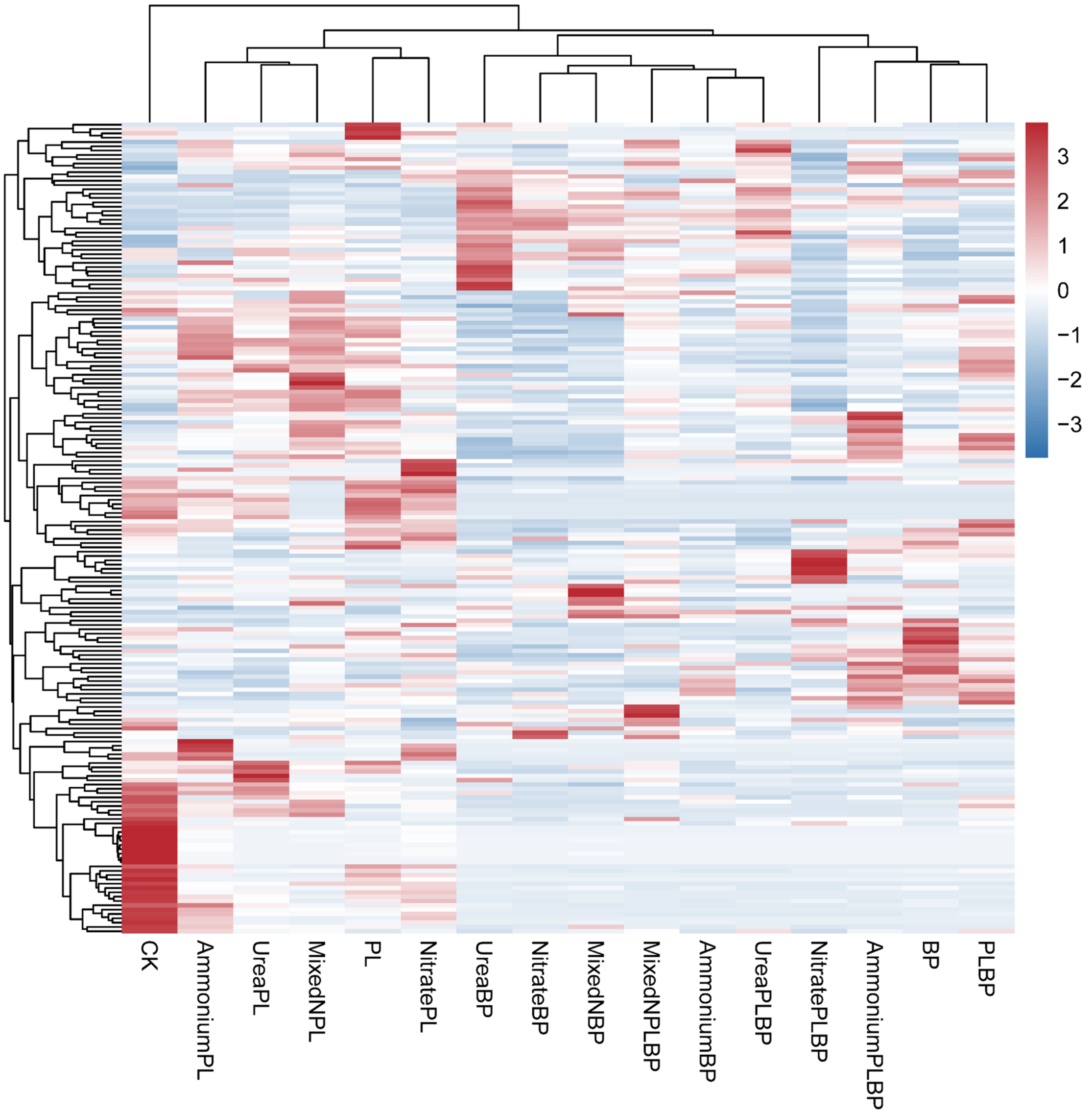

Figure S4 Heatmap of beta diversity estimates of soil bacterial communities based on the weighted UniFrac distance at the family level. The color blocks represent the distance values. In particular, the blue block denotes a relatively short distance between the samples, as well as a high degree of similarity. Conversely, the red block denotes a greater distance between the samples, as well as a low degree of similarity. Abbreviations: CK, control; Ni, nitrate; Am, ammonium; Ur, urea; MixedN, mixed nitrogen; PL, monocultural *Pterocypsela laciniata* (Houtt.) Shih; BP, monocultural *Bidens pilosa* L.; PLBP, co-cultivated *Bidens pilosa* L. and *Pterocypsela laciniata* (Houtt.) Shih.

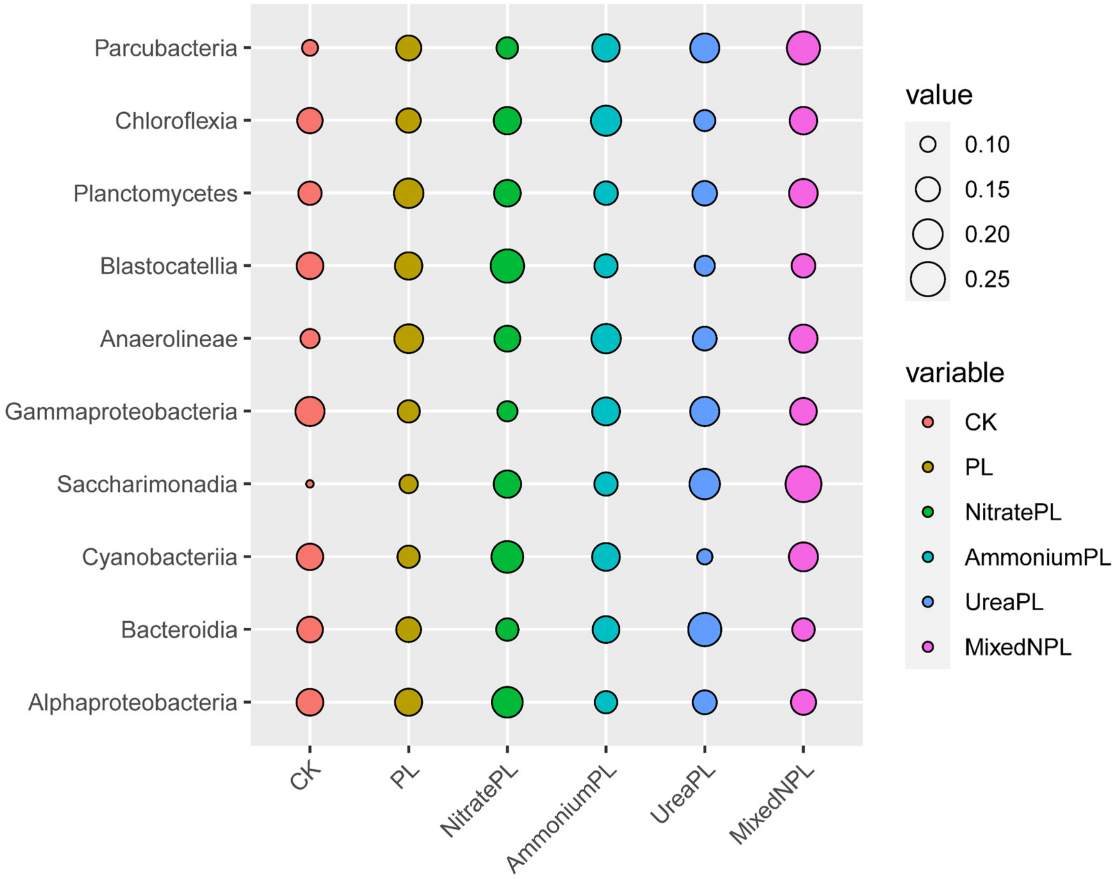

Parcubacteria
Chloroflexia
Planctomycetes
Blastocatellia
Anaerolineae
Gammaproteobacteria
Saccharimonadia
Cyanobacteriia
Bacteroidia
Alphaproteobacteria
CK
PL
NitratePL
AmmoniumPL
UreaPL
MixedNPL
value
0.10
0.15
0.20
0.25
variable
CK
PL
NitratePL
AmmoniumPL
UreaPL
MixedNPL

(a)

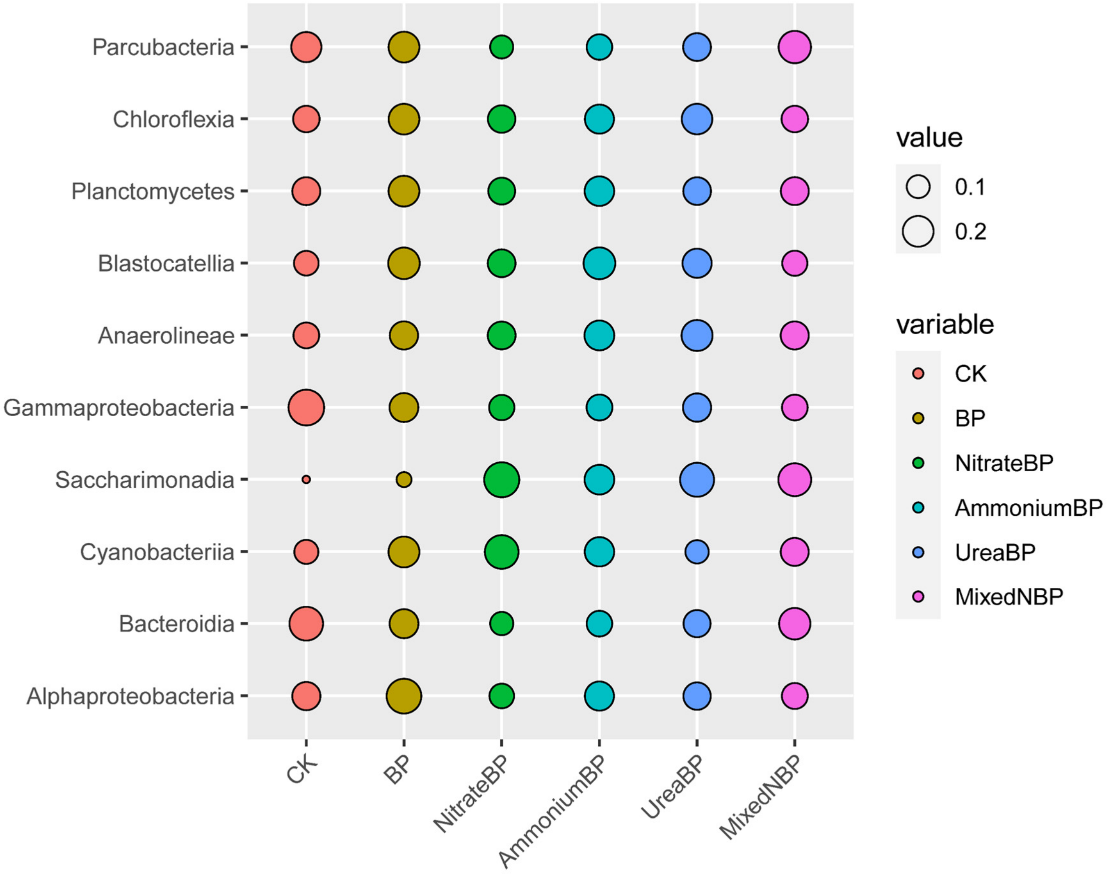
Parcubacteria
Chloroflexia
Planctomycetes
Blastocatellia
Anaerolineae
Gammaproteobacteria
Saccharimonadia
Cyanobacteriia
Bacteroidia
Alphaproteobacteria
CK
BP
NitrateBP
AmmoniumBP
UreaBP
MixedNBP
value
0.1
0.2
variable
CK
BP
NitrateBP
AmmoniumBP
UreaBP
MixedNBP

(b)

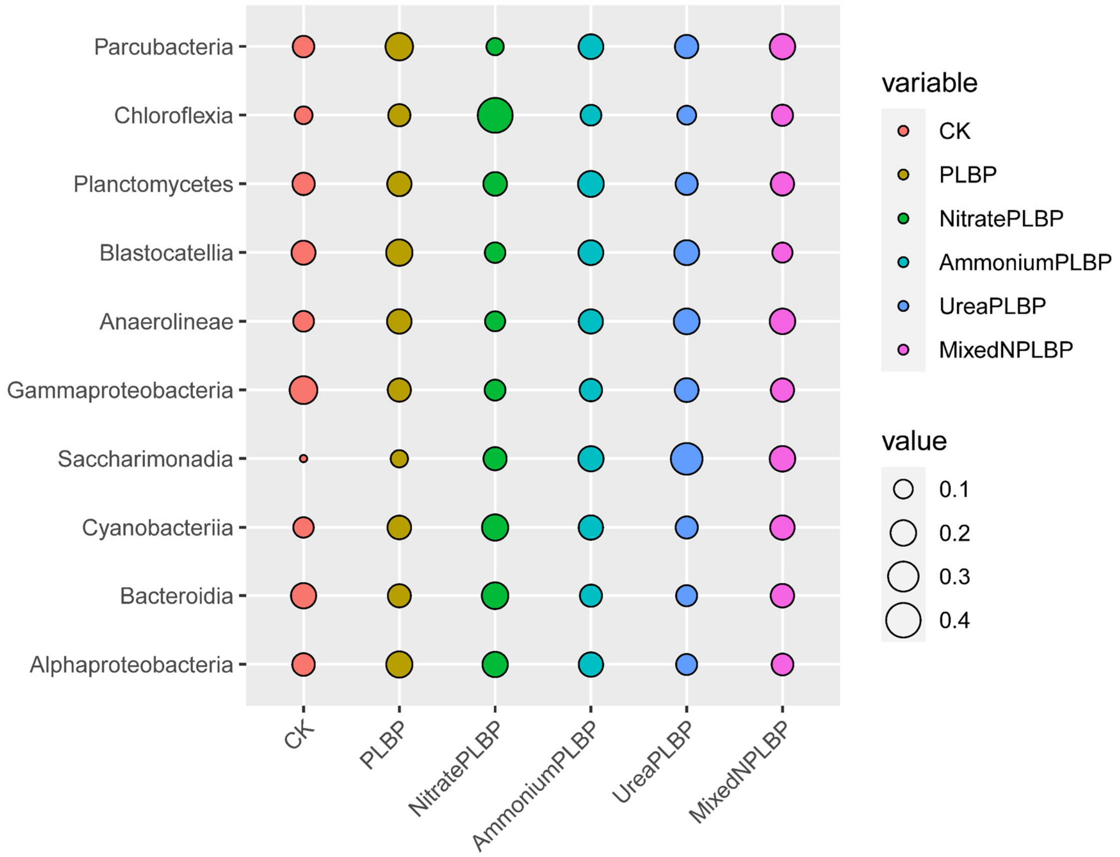

(c)

Figure S5 Bubble chart of soil bacterial biomarkers at the class level (**a**, monocultural *Pterocypsela laciniata* (Houtt.) Shih treated with artificially simulated nitrogen deposition with four forms; **b**, monocultural *Bidens pilosa* L. treated with artificially simulated nitrogen deposition with four forms; **c**, co-cultivated *Bidens pilosa* L. and *Pterocypsela laciniata* (Houtt.) Shih treated with artificially simulated nitrogen deposition with four forms). Abbreviations: CK, control; MixedN, mixed nitrogen; PL, monocultural *Pterocypsela laciniata* (Houtt.) Shih; BP, monocultural *Bidens pilosa* L.; PLBP, co-cultivated *Bidens pilosa* L. and *Pterocypsela laciniata* (Houtt.) Shih.

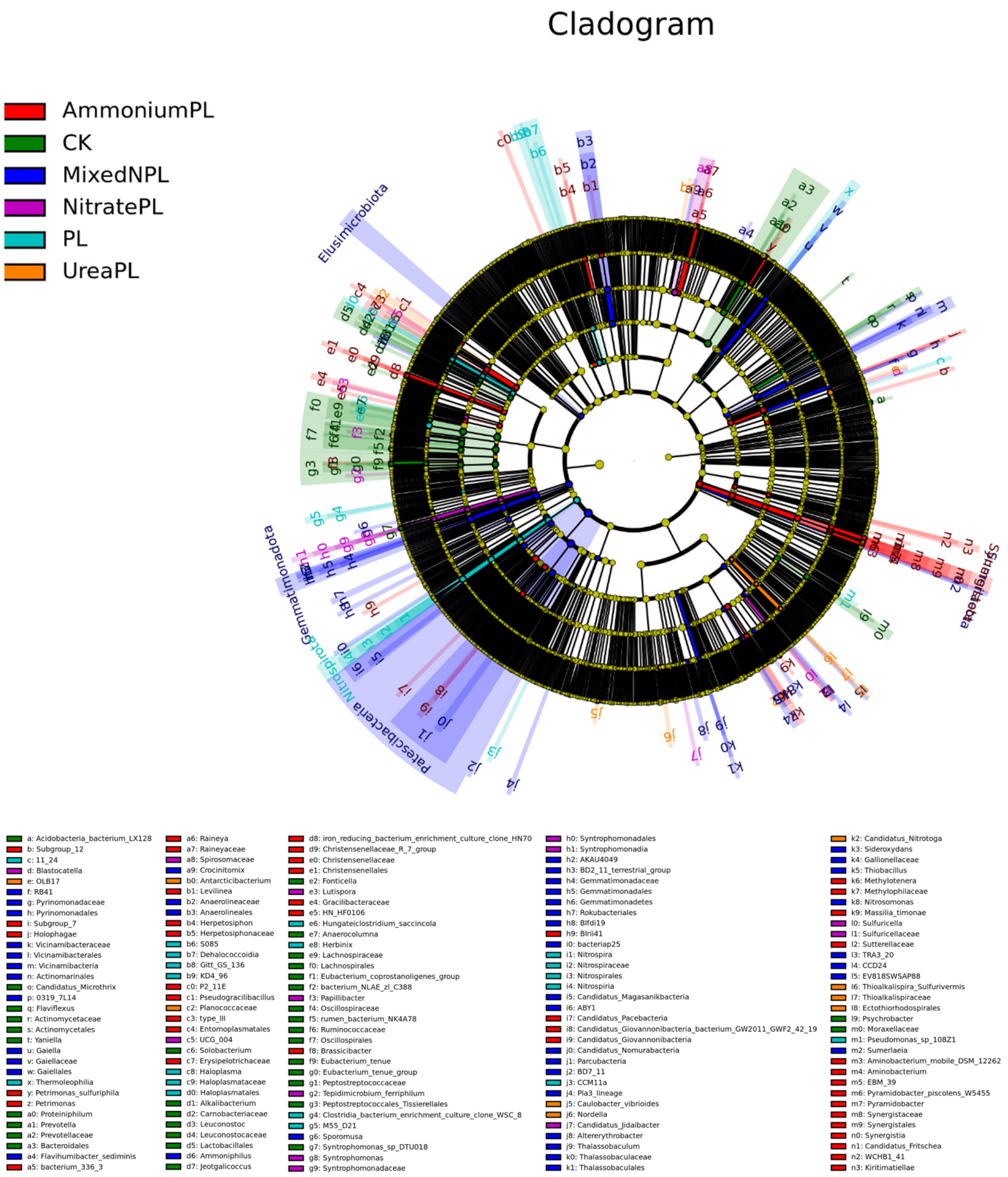

Cladogram
AmmoniumPL
CK
MixedNPL
NitratePL
PL
UreaPL
Elusimicrobiota
Patescibacteria
a: Acidobacteria_bacterium_LX128
b: Subgroup_12
c: 11_24
d: Blastocatella
e: OLB17
f: RB41
g: Pyrinomonadaceae
h: Pyrinomonadales
i: Subgroup_7
j: Holophagae
k: Vicinamibacteraceae
l: Vicinamibacterales
m: Vicinamibacteria
n: Actinomarinales
o: Candidatus_Microthrix
p: 0319_7L14
q: Flaviflexus
r: Actinomycetaceae
s: Actinomycetales
t: Yaniella
u: Gaiella
v: Gaiellaceae
w: Gaiellales
x: Thermoleophilia
y: Petrimonas_sulfuriphila
z: Petrimonas
a0: Proteiniphilum
a1: Prevotella
a2: Prevotellaceae
a3: Bacteroidales
a4: Flavihumibacter_sediminis
a5: bacterium_336_3
a6: Raineya
a7: Raineyaceae
a8: Spirosomaceae
a9: Crocinitomix
b0: Antarcticibacterium
b1: Levilinea
b2: Anaerolineaceae
b3: Anaerolineales
b4: Herpetosiphon
b5: Herpetosiphonaceae
b6: S085
b7: Dehalococcoidia
b8: Gitt_GS_136
b9: KD4_96
c0: P2_11E
c1: Pseudogracilibacillus
c2: Planococcaceae
c3: type_III
c4: Entomoplasmatales
c5: UCG_004
c6: Solobacterium
c7: Erysipelotrichaceae
c8: Haloplasma
c9: Haloplasmataceae
d0: Haloplasmatales
d1: Alkalibacterium
d2: Carnobacteriaceae
d3: Leuconostoc
d4: Leuconostocaceae
d5: Lactobacillales
d6: Ammoniphilus
d7: Jeotgalicoccus
d8: iron_reducing_bacterium_enrichment_culture_clone_HN70
d9: Christensenellaceae_R_7_group
e0: Christensenellaceae
e1: Christensenellales
e2: Fonticella
e3: Lutispora
e4: Gracilibacteraceae
e5: HN_HF0106
e6: Hungateiclostridium_saccincola
e7: Anaerocolumna
e8: Herbinix
e9: Lachnospiraceae
f0: Lachnospirales
f1: Eubacterium_coprostanoligenes_group
f2: bacterium_NLAE_zl_C388
f3: Papillibacter
f4: Oscillospiraceae
f5: rumen_bacterium_NK4A78
f6: Ruminococcaceae
f7: Oscillospirales
f8: Brassicibacter
f9: Eubacterium_tenue
g0: Eubacterium_tenue_group
g1: Peptostreptococcaceae
g2: Tepidimicrobium_ferriphilum
g3: Peptostreptococcales_Tissierellales
g4: Clostridia_bacterium_enrichment_culture_clone_WSC_8
g5: M55_D21
g6: Sporomusa
g7: Syntrophomonas_sp_DTU018
g8: Syntrophomonas
g9: Syntrophomonadaceae
h0: Syntrophomonadales
h1: Syntrophomonadia
h2: AKAU4049
h3: BD2_11_terrestrial_group
h4: Gemmatimonadaceae
h5: Gemmatimonadales
h6: Gemmatimonadetes
h7: Rokubacteriales
h8: Blfdi19
h9: Blrii41
i0: bacteriap25
i1: Nitrospira
i2: Nitrospiraceae
i3: Nitrospirales
i4: Nitrospiria
i5: Candidatus_Magasanikbacteria
i6: ABY1
i7: Candidatus_Pacebacteria
i8: Candidatus_Giovannonibacteria_bacterium_GW2011_GWF2_42_19
i9: Candidatus_Giovannonibacteria
j0: Candidatus_Nomurabacteria
j1: Parcubacteria
j2: BD7_11
j3: CCM11a
j4: Pla3_lineage
j5: Caulobacter_vibrioides
j6: Nordella
j7: Candidatus_Jidaibacter
j8: Altererythrobacter
j9: Thalassobaculum
k0: Thalassobaculaceae
k1: Thalassobaculales
k2: Candidatus_Nitrotoga
k3: Sideroxydans
k4: Gallionellaceae
k5: Thiobacillus
k6: Methylotenera
k7: Methylophilaceae
k8: Nitrosomonas
k9: Massilia_timonae
l0: Sulfuricella
l1: Sulfuricellaceae
l2: Sutterellaceae
l3: TRA3_20
l4: CCD24
l5: EV818SWSAP88
l6: Thioalkalispira_Sulfurivermis
l7: Thioalkalispiraceae
l8: Ectothiorhodospirales
l9: Psychrobacter
m0: Moraxellaceae
m1: Pseudomonas_sp_108Z1
m2: Sumerlaeia
m3: Aminobacterium_mobile_DSM_12262
m4: Aminobacterium
m5: EBM_39
m6: Pyramidobacter_piscolens_W5455
m7: Pyramidobacter
m8: Synergistaceae
m9: Synergistales
n0: Synergistia
n1: Candidatus_Fritschea
n2: WCHB1_41
n3: Kiritimatiellae

(a)

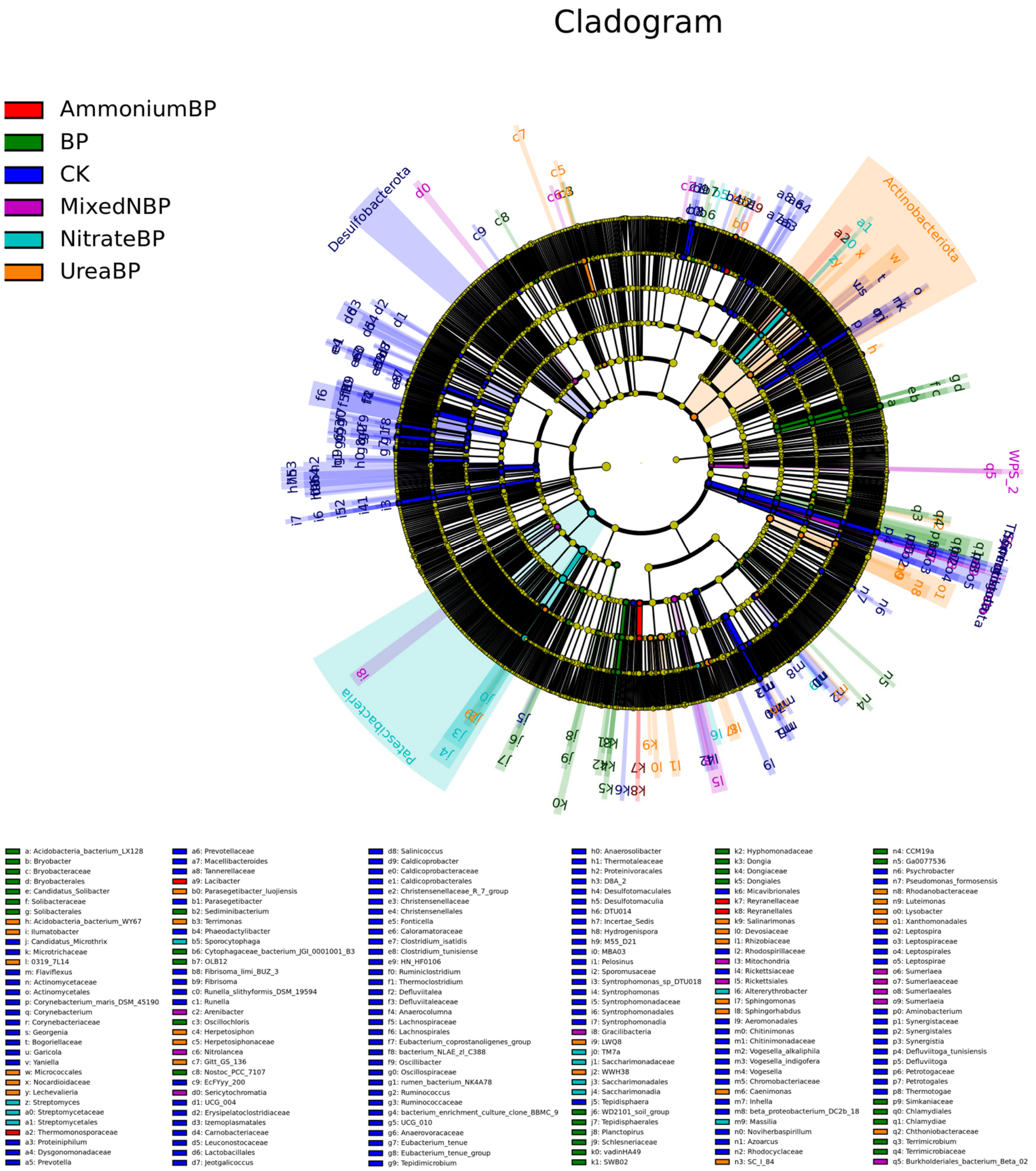
Cladogram
AmmoniumBP
BP
CK
MixedNBP
NitrateBP
UreaBP
Desulfobacterota
Actinobacteriota
Patescibacteria
a: Acidobacteria_bacterium_LX128
b: Bryobacter
c: Bryobacteraceae
d: Bryobacterales
e: Candidatus_Solibacter
f: Solibacteraceae
g: Solibacterales
h: Acidobacteria_bacterium_WY67
i: Ilumatobacter
j: Candidatus_Microthrix
k: Microtrichaceae
l: 0319_7L14
m: Flaviflexus
n: Actinomycetaceae
o: Actinomycetales
p: Corynebacterium_maris_DSM_45190
q: Corynebacterium
r: Corynebacteriaceae
s: Georgenia
t: Bogoriellaceae
u: Garicola
v: Yaniella
w: Micrococcales
x: Nocardioidaceae
y: Lechevalieria
z: Streptomyces
a0: Streptomycetaceae
a1: Streptomycetales
a2: Thermomonosporaceae
a3: Proteiniphilum
a4: Dysgonomonadaceae
a5: Prevotella
a6: Prevotellaceae
a7: Macellibacteroides
a8: Tannerellaceae
a9: Lacibacter
b0: Parasegetibacter_luojiensis
b1: Parasegetibacter
b2: Sediminibacterium
b3: Terrimonas
b4: Phaeodactylibacter
b5: Sporocytophaga
b6: Cytophagaceae_bacterium_JGI_0001001_B3
b7: OLB12
b8: Fibrisoma_limi_BUZ_3
b9: Fibrisoma
c0: Runella_slithyformis_DSM_19594
c1: Runella
c2: Arenibacter
c3: Oscillochloris
c4: Herpetosiphon
c5: Herpetosiphonaceae
c6: Nitrolancea
c7: Gitt_GS_136
c8: Nostoc_PCC_7107
c9: EcFYyy_200
d0: Sericytochromatia
d1: UCG_004
d2: Erysipelatoclostridiaceae
d3: Izemoplasmatales
d4: Carnobacteriaceae
d5: Leuconostocaceae
d6: Lactobacillales
d7: Jeotgalicoccus
d8: Salinicoccus
d9: Caldicoprobacter
e0: Caldicoprobacteraceae
e1: Caldicoprobacterales
e2: Christensenellaceae_R_7_group
e3: Christensenellaceae
e4: Christensenellales
e5: Fonticella
e6: Caloramatoraceae
e7: Clostridium_isatidis
e8: Clostridium_tunisiense
e9: HN_HF0106
f0: Ruminiclostridium
f1: Thermoclostridium
f2: Defluviitalea
f3: Defluviitaleaceae
f4: Anaerocolumna
f5: Lachnospiraceae
f6: Lachnospirales
f7: Eubacterium_coprostanoligenes_group
f8: bacterium_NLAE_zl_C388
f9: Oscillibacter
g0: Oscillospiraceae
g1: rumen_bacterium_NK4A78
g2: Ruminococcus
g3: Ruminococcaceae
g4: bacterium_enrichment_culture_clone_BBMC_9
g5: UCG_010
g6: Anaerovoracaceae
g7: Eubacterium_tenue
g8: Eubacterium_tenue_group
g9: Tepidimicrobium
h0: Anaerosolibacter
h1: Thermotaleaceae
h2: Proteinivoracales
h3: D8A_2
h4: Desulfotomaculales
h5: Desulfotomaculia
h6: DTU014
h7: Incertae_Sedis
h8: Hydrogenispora
h9: M55_D21
i0: MBA03
i1: Pelosinus
i2: Sporomusaceae
i3: Syntrophomonas_sp_DTU018
i4: Syntrophomonas
i5: Syntrophomonadaceae
i6: Syntrophomonadales
i7: Syntrophomonadia
i8: Gracilibacteria
i9: LWQ8
j0: TM7a
j1: Saccharimonadaceae
j2: WWH38
j3: Saccharimonadales
j4: Saccharimonadia
j5: Tepidisphaera
j6: WD2101_soil_group
j7: Tepidisphaerales
j8: Planctopirus
j9: Schlesneriaceae
k0: vadinHA49
k1: SWB02
k2: Hyphomonadaceae
k3: Dongia
k4: Dongiaceae
k5: Dongiales
k6: Micavibrionales
k7: Reyranellaceae
k8: Reyranellales
k9: Salinarimonas
l0: Devosiaceae
l1: Rhizobiaceae
l2: Rhodospirillaceae
l3: Mitochondria
l4: Rickettsiaceae
l5: Rickettsiales
l6: Altererythrobacter
l7: Sphingomonas
l8: Sphingorhabdus
l9: Aeromonadales
m0: Chitinimonas
m1: Chitinimonadaceae
m2: Vogesella_alkaliphila
m3: Vogesella_indigofera
m4: Vogesella
m5: Chromobacteriaceae
m6: Caenimonas
m7: Inhella
m8: beta_proteobacterium_DC2b_18
m9: Massilia
n0: Noviherbaspirillum
n1: Azoarcus
n2: Rhodocyclaceae
n3: SC_I_84
n4: CCM19a
n5: Ga0077536
n6: Psychrobacter
n7: Pseudomonas_formosensis
n8: Rhodanobacteraceae
n9: Luteimonas
o0: Lysobacter
o1: Xanthomonadales
o2: Leptospira
o3: Leptospiraceae
o4: Leptospirales
o5: Leptospirae
o6: Sumerlaea
o7: Sumerlaeaceae
o8: Sumerlaeales
o9: Sumerlaeia
p0: Aminobacterium
p1: Synergistaceae
p2: Synergistales
p3: Synergistia
p4: Defluviitoga_tunisiensis
p5: Defluviitoga
p6: Petrotogaceae
p7: Petrotogales
p8: Thermotogae
p9: Simkaniaceae
q0: Chlamydiales
q1: Chlamydiae
q2: Chthoniobacteraceae
q3: Terrimicrobium
q4: Terrimicrobiaceae
q5: Burkholderiales_bacterium_Beta_02

(b)

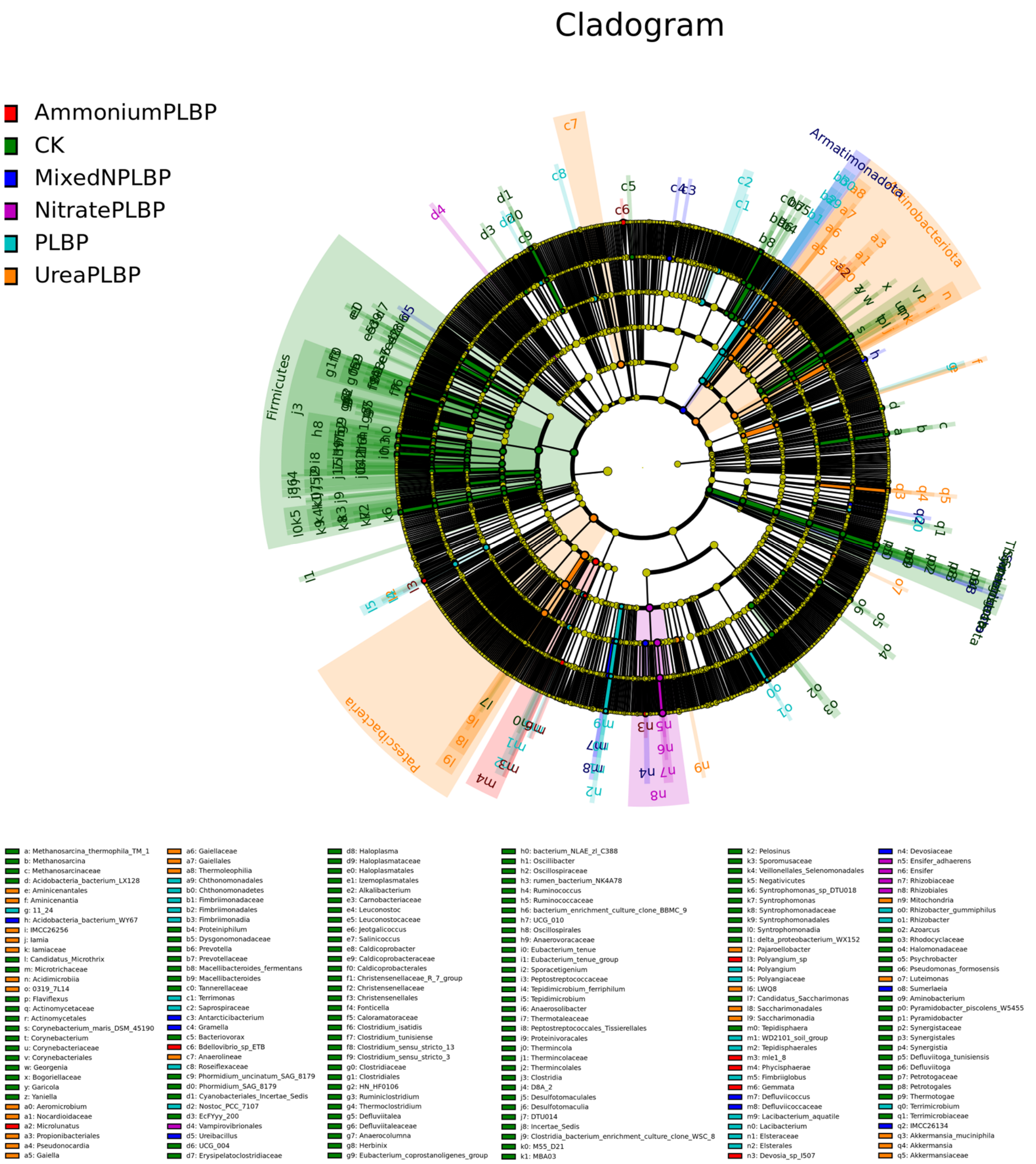

(c)

Figure S6 LEfSe method identifies the significantly different abundant taxa of soil bacterial communities with different treatments (**a**, monocultural *Pterocypsela laciniata* (Houtt.) Shih treated with artificially simulated nitrogen deposition with four forms; **b**, monocultural *Bidens pilosa* L. treated with artificially simulated nitrogen deposition with four forms; **c**, co-cultivated *Bidens pilosa* L. and *Pterocypsela laciniata* (Houtt.) Shih treated with artificially simulated nitrogen deposition with four forms). The taxa with significantly different abundances among treatments are

indicated by coloured dots, which characterise the kingdom, phylum, class, order, family, genus, and species levels, respectively, from the centre outward. The coloured shadows are indicative of the trends observed in the significantly different taxa. Only those taxa that meet an LDA significance threshold of >2 are shown. Abbreviations: CK, control; MixedN, mixed nitrogen; PL, monocultural *Pterocypsela laciniata* (Houtt.) Shih; BP, monocultural *Bidens pilosa* L.; PLBP, co-cultivated *Bidens pilosa* L. and *Pterocypsela laciniata* (Houtt.) Shih.
